# Disturbance study of seismic vibrator reaction mass and piston

**DOI:** 10.1371/journal.pone.0225259

**Published:** 2019-12-05

**Authors:** Zhen Chen, Zhiqiang Huang, Shuang Jing, Yang Zhou, Yan Chen, Hongyang Zeng

**Affiliations:** 1 MOE Key Lab of Oil and Natural Gas Equipment, School of Mechatronic Engineering, Southwest Petroleum University, Chengdu, China; 2 College of Petroleum Engineering, Southwest Petroleum University, Chengdu, China; 3 Hunan Jiangli Rongda Vehicle Transmission Limited Company, Changsha, Hunan, China; Northeastern University, UNITED STATES

## Abstract

The seismic vibrator has become a main energy source for land seismic exploration. For a seismic vibrator, a piston is installed in the vertical direction in the reaction mass, and the reaction mass moves up and down along the piston. Because of vibrator dynamic disturbances in the reaction mass and baseplate system, the vibrator output force is contaminated by harmonic distortion. This distorted output force results in poor quality of seismic data. This paper studies the dynamics and disturbances of the piston as well as the reaction mass. The results show that the reaction mass disturbance is mainly caused by the horizontal oil flow inlet going to the reaction mass chamber. Precisely speaking, the ideal vertical motion of the reaction mass is disturbed by a combination of a reaction mass rotational motion along the piston and a bending motion of the piston. Changing the oil flow inlet from the horizontal direction to the vertical direction significantly reduces these disturbances. In the disturbance test of the vertical oil flow inlet, the disturbance of the reaction mass in XOZ and YOZ is reduced by 73.943% and 54.232%, respectively, and the disturbance of the vibrator is obviously weakened. The motion of the reaction mass is close to the ideal vertical direction. This change of the oil flow inlet direction further improves the accuracy and reliability of the vibrator output force.

## 1. Introduction

Hydraulic disturbance is one of the key factors that limits the operational frequency range of the vibrator vibration. This disturbance also causes the resonances of the trapped oil in the reaction mass chamber and flow passages. The disturbance further reduces the life cycle of hydraulic components, leading to high operational failure rates. For example, hydraulic disturbance causes severe oil leakage of vibrator accumulators and burns the servo-valve assembly [1–3]. Improving hydraulic oil flow disturbance rejection in the reaction mass chamber and its passages can increase the signal-to-noise ratio in the vibrator output force signal. Eventually, this improved vibrator output force signal will be efficiently transmitted into the ground, and it results in better seismic data records [4–7]. Therefore, accurately understanding the disturbances that occur in the reaction mass chamber becomes very necessary.

Increasing the signal-to-noise ratio of the vibrator output force can be interpreted as the output force signal having an improved amplitude and phase with much less harmonic distortion. Improving the ratio also means that finding solutions to reduce disturbances as well as harmonic distortion becomes very critical in vibroseis acquisition [8]. Many researchers have done great work to identify the root causes of, for example, the interference among vibrators [9], the vibrator hydraulic supply pressure fluctuations [10–11], the baseplate decoupling [11–15] and the nonlinear behaviors of the servo-valve assembly [11, 13, 15, 16, 17,18]. Meanwhile, some researchers have carried out the research work on the hydraulic system disturbance and the water hammer. In 2010, Min Li[19] carried out the researches of the numerical investigation on water hammer process of viscoelastic fluid variable frequency flow regulation systems. The influencing rules of viscoelastic rheological parameters on the water hammer were modeled by the stable numerical algorithm. The simulation results show that water hammer strength increase with increasing of viscoelastic fluid relaxation time and density, but decrease with increasing of viscoelastic fluid viscosity. In 2011, Lingyan Zhang[20] did some research on the correlating control model of water hammer caused by variable frequency flow regulation, the correlating control model of the rheological properties parameters which avoided water hammer caused by variable frequency flow regulation was established. The research results lay theoretical foundation for water hammer pressure precise control. In 2009, Bin Chen[21] carried out the mechanism of coupled nonlinear vibration phenomenon, by taking a certain type hydraulic steering gear control valve as example. The results may provide theoretical basis for understanding the vibration characteristics of hydraulic system and its control measures, also for hydraulic valve design and improvement. In 2007, Pan Xudong[22] studied the flow characteristics of the three-stage electro-hydraulic servo valve power stage ideal spool valve through FLUENT simulation technology, and obtained the flow coefficient characteristic curve of the three-stage electro-hydraulic servo valve power stage ideal spool valve. In 2006, Yang chao [23] carried out the analysis of hydraulic-water hammer induced axial vibration response based on FSI model, and established fluid-solid interaction (FSI) 4-equation model. Through theoretical and experimental methods, it is found that the surge pressure decreases with the increase of the pipe Poisson’s ratio and increases with the increase of the pipe wall thickness, to guide the design of the hydraulic system pipeline.

However, few studies are reported on the impact of the vibrator output force due to disturbances of the reaction mass and piston bending. This paper attempts to fill that gap. It is organized as follows. First, the disturbance that occurred in the reaction mass is presented. Then, mechanical models of the piston and hydraulic oil flow passages are built and illustrated. Next, dynamic simulations based on those mechanical models are performed to find the cause of the disturbance. An optimal position of hydraulic oil flow inlet is proposed for suppressing the disturbances. Finally, experimental tests are carried out, and the results are presented for verification.

Based on this conclusion and combined with the reaction mass trajectory test, in this paper, the dynamic behavior law of the reaction mass—piston rod by theoretical calculation and numerical simulation based on the vibrator horizontal channel has been studied; the impact of the horizontal passageway on the reaction mass movement and the piston rod deformation is analyzed, and a direct cause of the large disturbance of the vibrator is found. According to the analysis results, a vertical passageway vibrator has been proposed and compared with the results of the horizontal passageway. The results show that the vertical passageway can effectively reduce the disturbance of the vibrator, which can provide guidance for the development of high-quality and high-resolution vibroseis.

## 2. Disturbances in the reaction mass system

**Fig 1:**
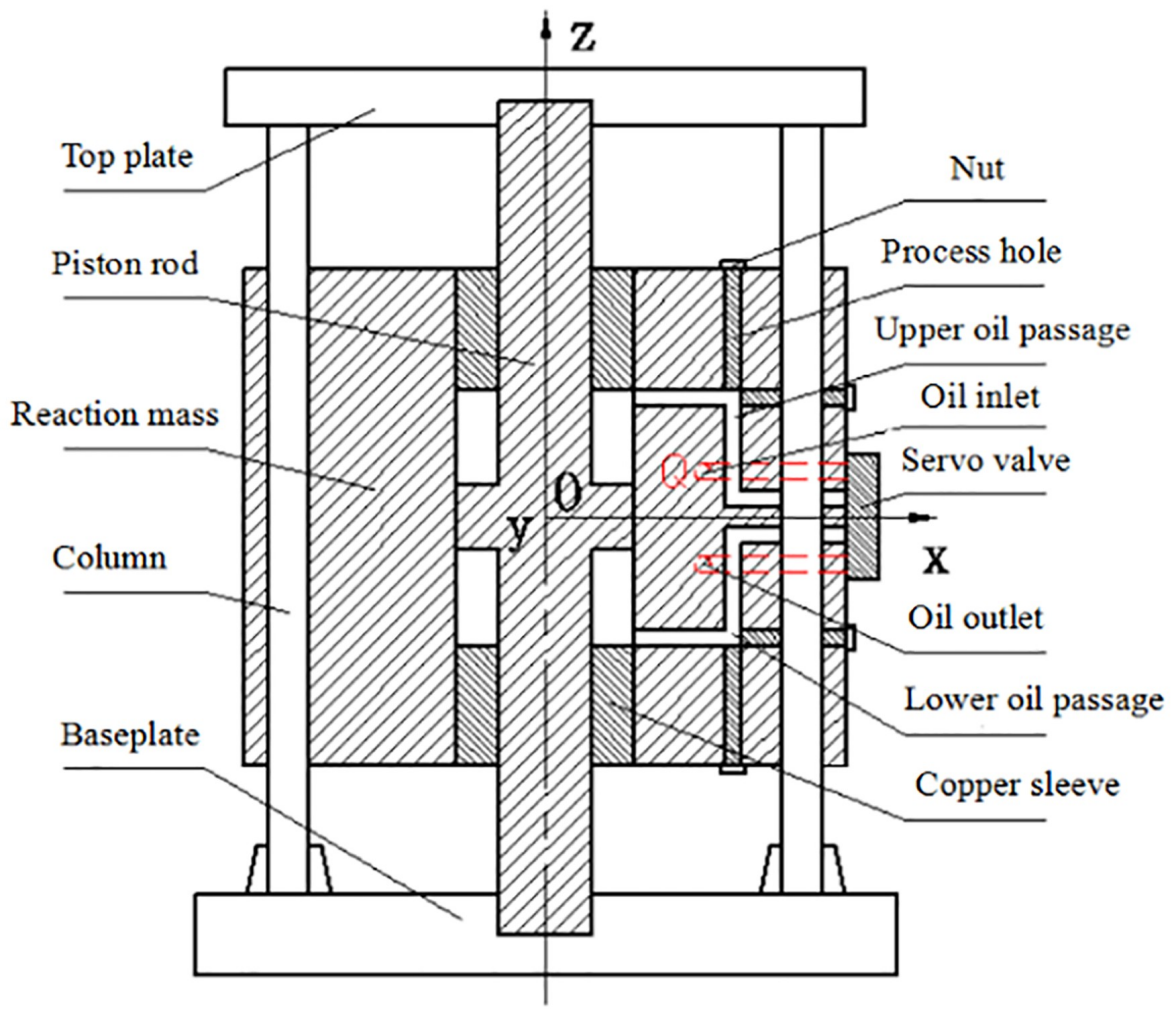
Simplified structure of horizontal oil inlet vibrator.

A modern seismic vibrator is essentially a hydromechanical system driven by a servo-valve assembly that is controlled electronically. Fig 1 depicts a cross-section of the reaction mass and baseplate assembly of a BGP K-type vibrator. The piston is rigidly connected to the top plate and the bottom baseplate. The top plate and the bottom baseplate are firmly bolted with four columns. The piston, the top plate, the bottom baseplate and four columns are mechanically integrated together and form a vibrator driven structure. A cylindrical bore is machined inside the reaction mass such that the piston divides the cylindrical bore into an upper chamber and a lower chamber. Two oil passages are machined in one sidewall of the reaction mass and connected to two control ports of the servo-valve assembly. High-pressure hydraulic oil flows out of the servo-valve assembly and is fed alternately into the upper and lower chambers to drive the reaction mass to move up and down along the piston. The force acting on the reaction mass is equally and oppositely applied to the piston. Eventually, the force is radiated into the ground by the bottom baseplate. This force is called the vibrator ground force.

### 2.1 Turbulence model in reaction mass

The hydraulic oil in a hydraulic pipeline of the vibrator reaction mass is an incompressible fluid, and the flow state of the oil is a highly complex three-dimensional unsteady flow at a high-speed reversal time of the servo valve. In engineering, the Navier-Stokes (N-S) equation is usually used to describe a turbulent flow. The time-averaged control model is given by [24]:

Continuity equation:

$$\frac{\partial u_{i}}{\partial x_{i}}=0$$ (1)

Momentum equation:

$$\rho \frac{\partial u_{i}}{\partial t}+\rho \frac{\partial \left(u_{i}u_{j}\right)}{\partial x_{j}}=-\frac{\partial p}{\partial x_{i}}+\frac{\partial }{\partial x_{j}}(\mu \frac{\partial u_{i}}{\partial x_{j}}-\rho \bar{{u^{\prime }}_{i}{u^{\prime }}_{j}})$$ (2)

where *ρ* is the density of the flow, *t* is the time, *x*_*i*_ is the spatial coordinate along the x direction (*i* = 1, 2, 3), *u*_*i*_ is the time-averaged velocity along the *i* direction, *p* is the pressure, and *μ* is the time-averaged viscosity.

The Reynolds stress equation is given by

$$-\rho \bar{{u^{\prime }}_{i}{u^{\prime }}_{j}}=\mu_{t}(\frac{\partial u_{i}}{\partial x_{j}}+\frac{\partial u_{j}}{\partial x_{i}})-\frac{2}{3}(\rho k+\mu_{t}\frac{\partial u_{i}}{\partial x_{i}})\delta_{ij}$$ (3)

where *k* is the turbulent kinetic energy, *u*_*t*_ is the turbulent viscosity coefficient, and *δ*_*ij*_ is the Kronecker function specified as

$$\{\begin{array}{c} \delta_{ij}=1(i=j)\\ \delta_{ij}\neq 1(i\neq j) \end{array}$$ (4)

The equations for turbulent kinetic energy *k* and dissipation rate *ε* for steady incompressible fluids are given by

$$\rho u_{i}\frac{\partial k}{\partial x_{i}}=\frac{\partial }{\partial x_{i}}\left[(\mu +\frac{\mu_{t}}{\sigma_{k}})\frac{\partial k}{\partial x_{i}}\right]+G_{k}-\rho \varepsilon$$ (5)

$$\rho \mu_{i}\frac{\partial \varepsilon }{\partial x_{i}}=\frac{\partial }{\partial x_{i}}\left[(\mu +\frac{\mu_{t}}{\sigma_{\varepsilon }})\frac{\partial \varepsilon }{\partial x_{i}}\right]+c_{1}\frac{\varepsilon }{k}G_{k}-c_{2}\rho \frac{\varepsilon^{2}}{k+\sqrt{v\varepsilon }}$$ (6)

where *G*_*k*_ is the stress source term.

$$G_{k}=\mu_{t}S^{2}$$ (7)

The turbulent kinetic energy *k* and dissipation rate *ε* represent the energy consumption level and signal difference in the vibrator that are caused by the turbulence from the high-frequency commutation of the vibrator. *k* and *ε* are highly related to the characteristic parameters of the hydraulic oil flow field, such as the force of the piston rod, flow field trace, pressure loss and turbulence intensity.

### 2.2 The reaction mass disturbances

**Fig 2:**
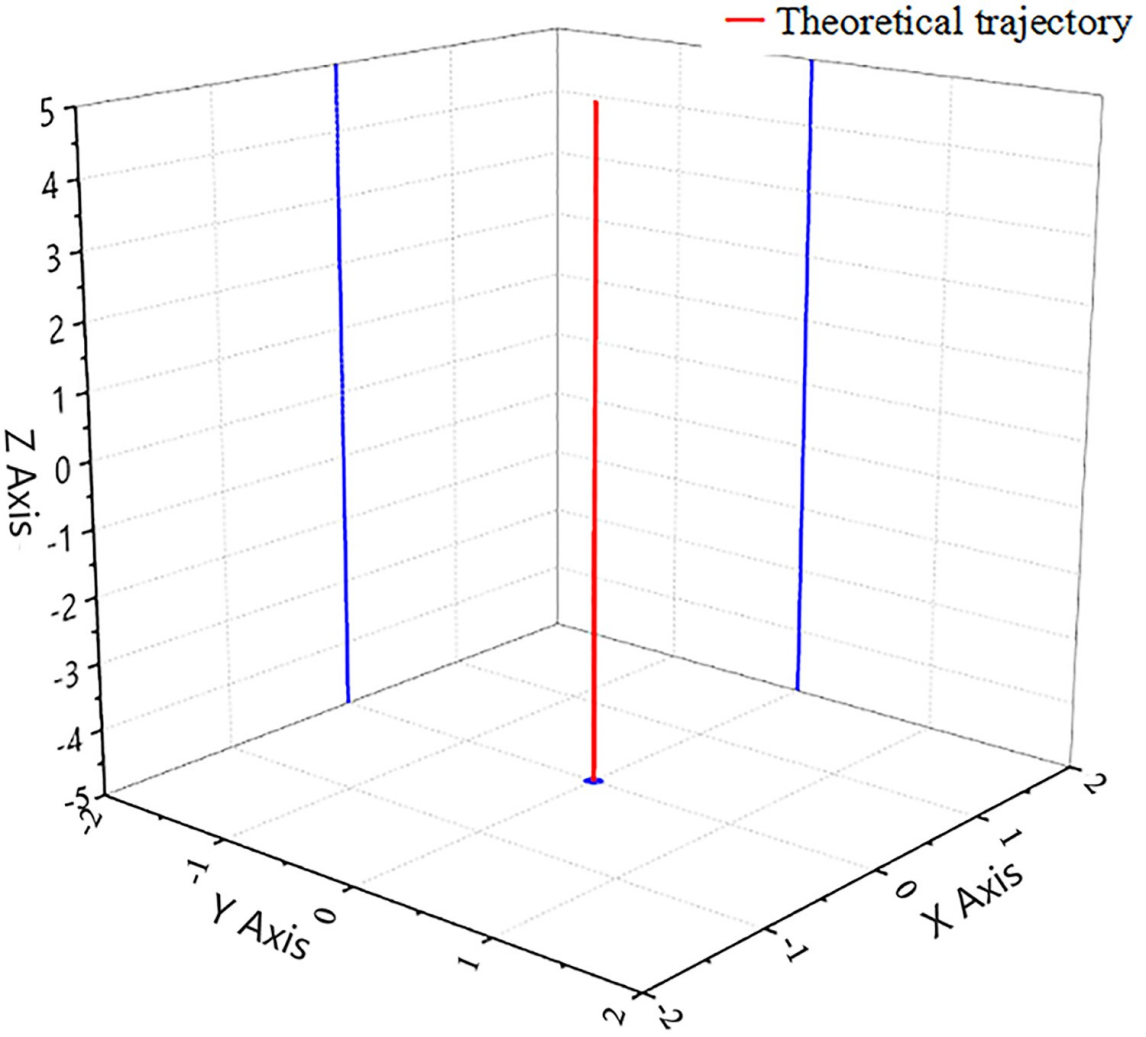
The ideal reaction mass movement path.

Fig 2 illustrates an ideal vibration displacement of the reaction mass. Theoretically, the reaction mass should move up and down in a straight vertical direction or a Z-axis direction. If the vibrator driven structure is a rigid body, no bending displacements should appear during vibrator vibration. However, in reality, due to the low rigidity of the vibrator-driven structure and the horizontal oil flow inlet of the reaction mass, the reaction mass motion displacement suffers from deviations from the ideal vertical direction. The more the reaction mass deviates from the vertical direction or becomes a space curve, the larger the disturbance quantity of the vibrator will be, the more the output signal quality will be influenced, and the more the distortion signal will be increased.

**Fig 3:**
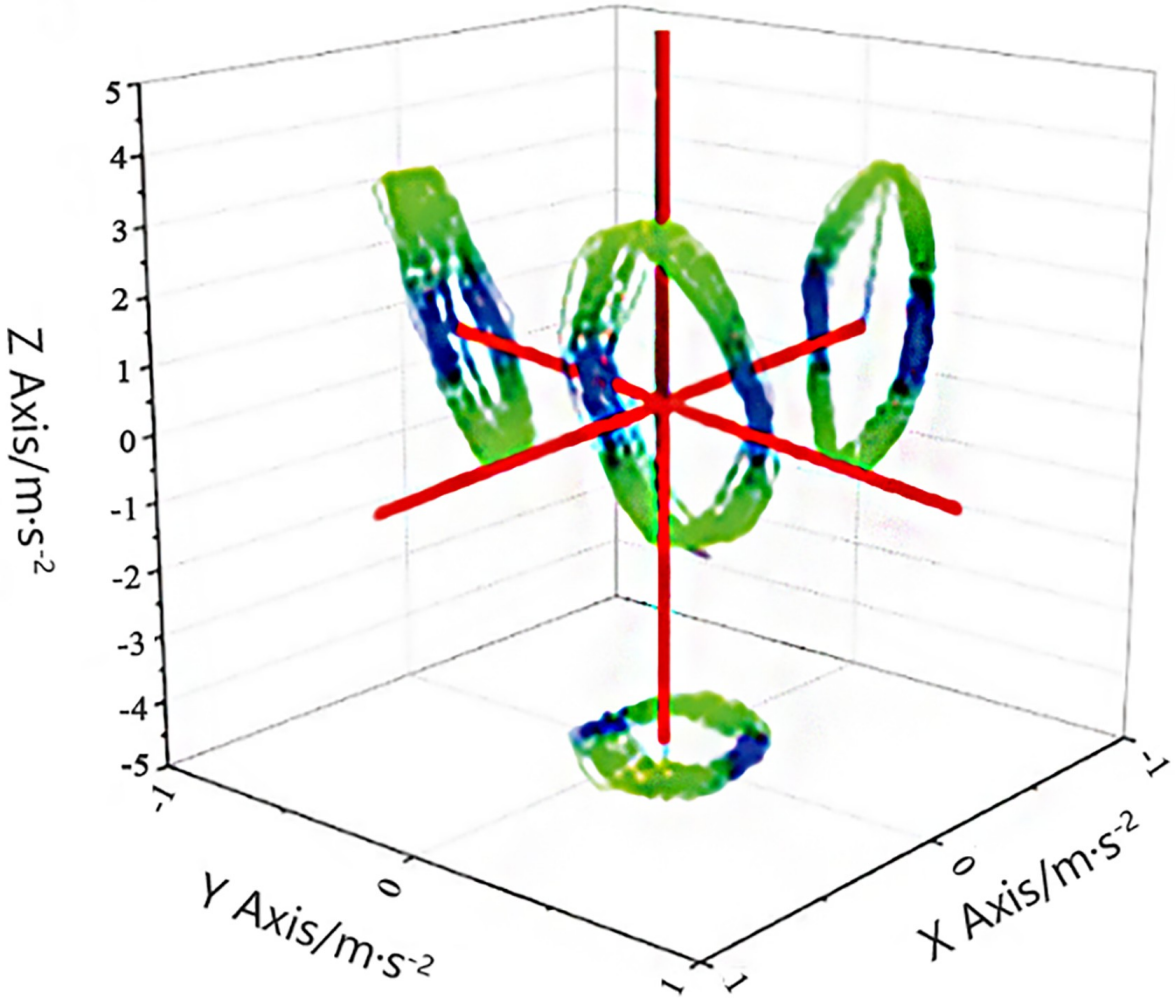
Motion disturbances of the reaction mass.

Fig 3 clearly illustrates these motion disturbances.

## 3. Mechanical simulation models

**Fig 4:**
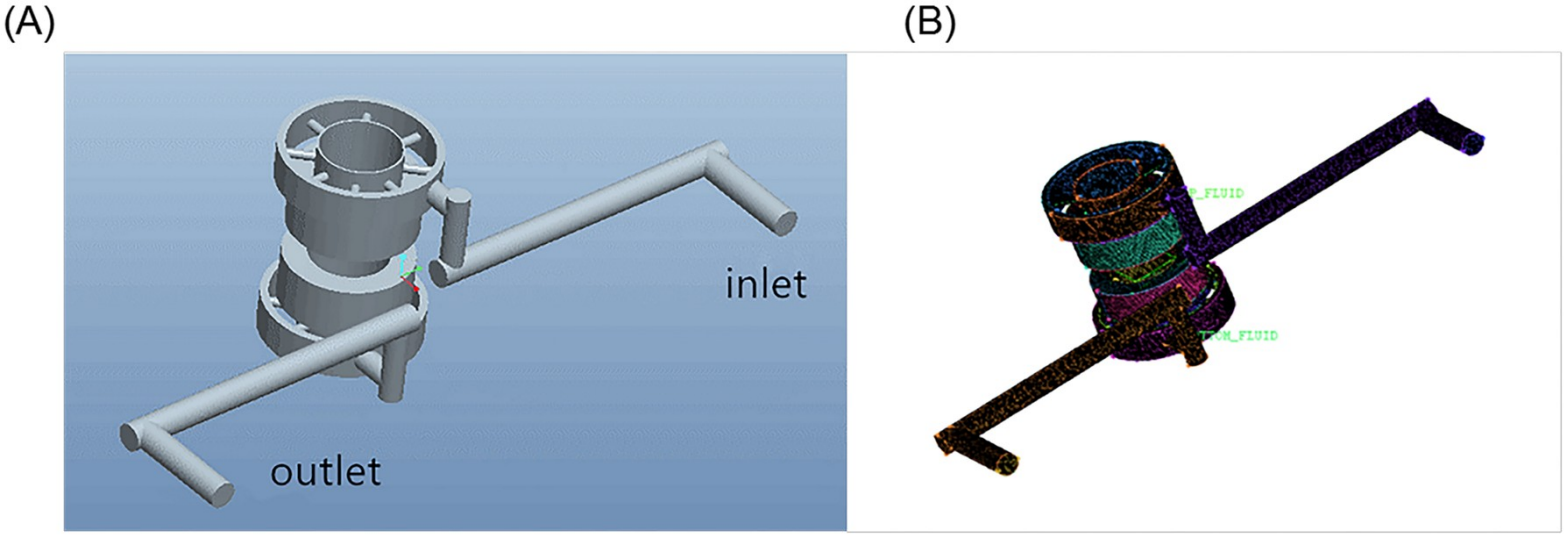
A mechanical fluid model of the hydraulic oil flow passage (left) and its meshed model (right). (A) fluid model (B) meshed model.

Because of low rigidity of the vibrator-driven structure, the disturbance caused by the hydraulic flow trapped in the reaction mass chamber and passages cannot be ignored. The left plot shown in Fig 4 depicts a mechanical fluid model of hydraulic oil flow passages. The pressurized hydraulic oil is fed through the inlet and flows out from the outlet. This flow will create a differential pressure across the piston, resulting in a force to move the reaction mass. The inlet and outlet passages are machined in the horizontal plane so that the hydraulic oil flow disturbance can be minimized [25], [26]. Finite element analysis tools such as computational fluid dynamics (CFD) and Solidworks Cosmos (CSM) [27] are used to simulate the dynamic responses of the mechanical fluid model. The right plot shown in Fig 4 shows a meshed mechanical fluid model of the hydraulic oil flow passage. Eventually, a numerical simulation of deformation for the oil flow field is calculated [28].

To analyze the influence of the horizontal inlet on the reaction mass movement, the numerical simulation of the hydraulic flow field of the reaction mass is established and the corresponding data are analyzed. Fluid geometry and grid models are shown in Fig 4.

**Fig 5:**
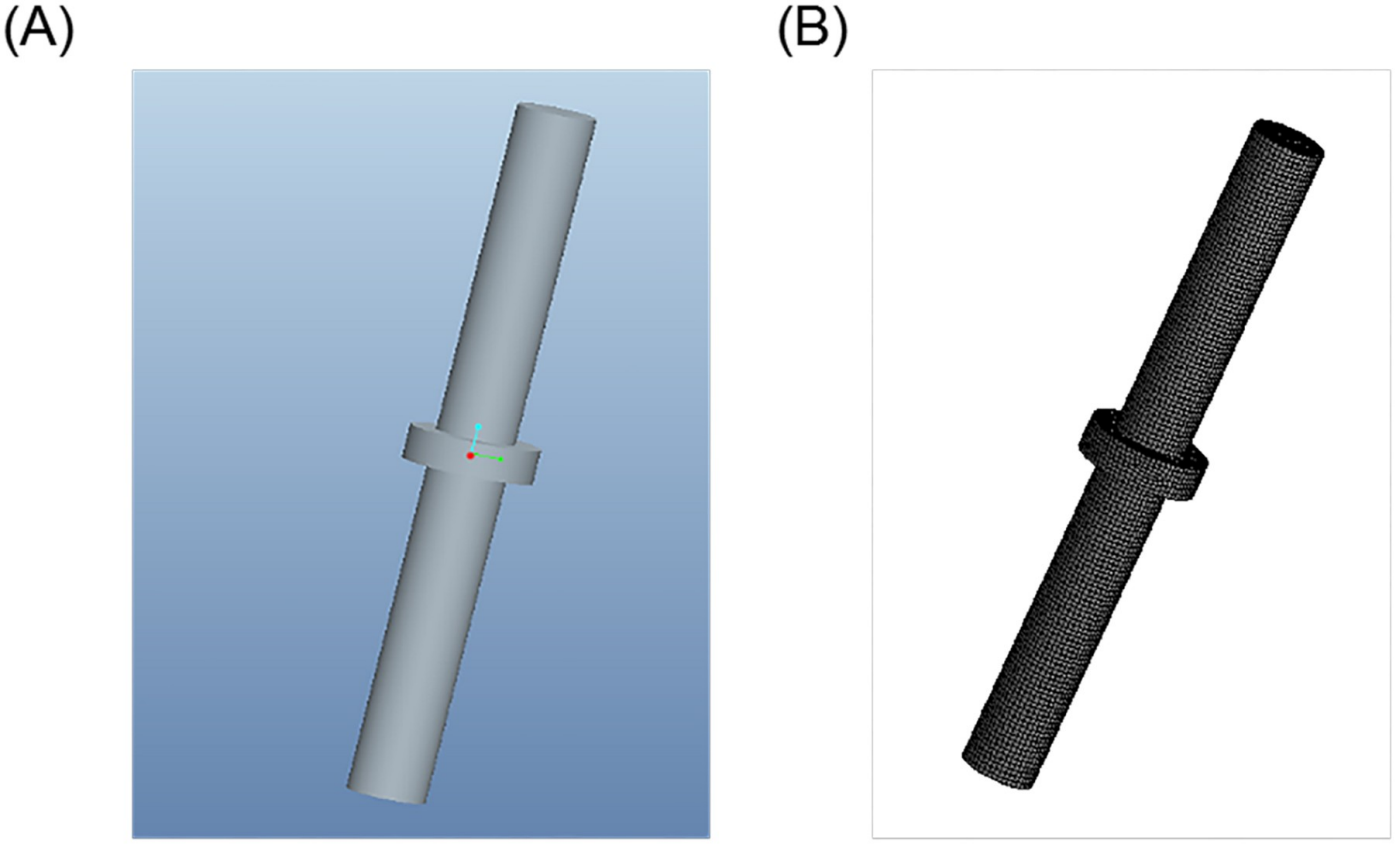
Piston rod geometry and grid model. (A) Piston rod model (B) Grid meshing.

Fig 5 shows the mechanical model of the piston (left) as well as its meshed model (right). The pressure data of the hydraulic oil flow calculated from the simulation shown in Fig 4 are applied to the piston area in the meshed model. Thus, the deformation of the piston can be obtained.

## 4. Simulation and results

To study disturbances using simulation models, necessary boundary conditions are needed. A differential pressure that is applied to the piston area in the reaction mass chamber is expressed by Eq 8.

Pd=17.5sin(2πf) (8)

where *Pd* is the differential pressure (MPa) and *f* is the frequency (Hz). Precisely speaking, in the positive half cycle, the differential pressure goes to the hydraulic oil inlet while a constant pressure that is equal to 0.62 MPa is applied to the hydraulic oil outlet. The hydraulic force is applied to the upper end face of the piston rod. In the negative half cycle, the differential pressure is altered to the hydraulic oil outlet while the constant pressure (0.62 MPa) goes to the hydraulic oil inlet S1 Fig. The hydraulic force is applied to the lower end face of the piston rod and is expressed by Eq 8.

A fixed constraint is applied to both the upper and lower ends of the piston. A fluid-solid coupling surface is chosen for the contact surface between the piston and the hydraulic fluid in the reaction mass chamber. Furthermore, a force of 19,144 N is loaded on the fluid-solid coupling surface along the X-direction, this force is calculated by the equation *F* = *P*·*S*, where, *P* is the hydraulic oil pressure, *S* is the area value of the hydraulic oil passages.

**Fig 6:**
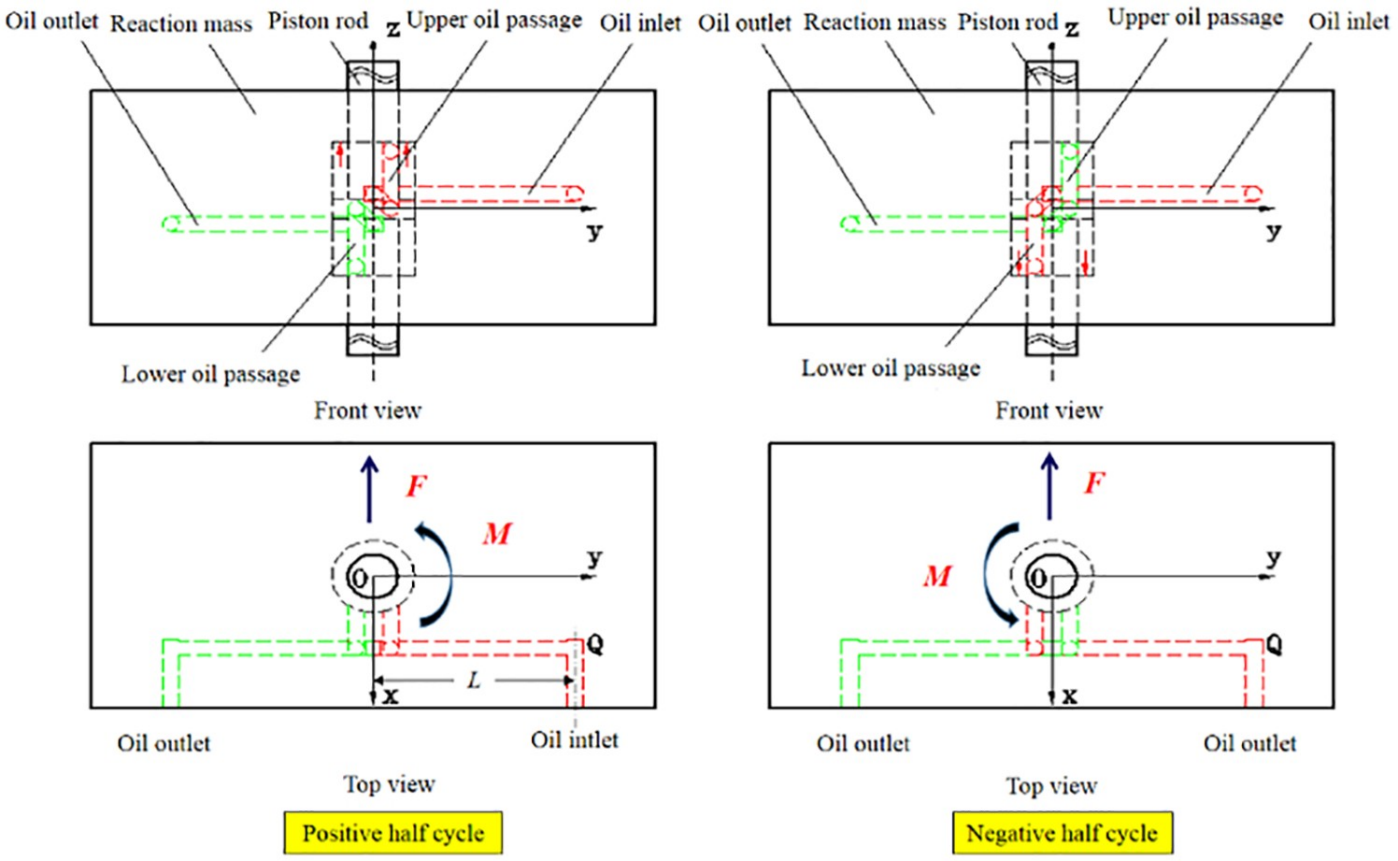
Connections between the reaction mass chambers and hydraulic oil passages.

As stated earlier, the differential pressure is alternately fed into the upper and lower chambers in the reaction mass through hydraulic oil passages. Fig 6 illustrates this connection. The hydraulic oil inlet is highlighted in red and fed with an oil flow at a pressure of 17.5 MPa, and the hydraulic oil outlet is at a pressure of 0.62 MPa and marked in green. It can be seen from the bottom graph in Fig 6 that a force will be produced at the right-angle corner marked as Q as the hydraulic oil flows through the oil inlet. This force can be theoretically calculated as a value of 19,144 N in the negative X-direction and causes a bending motion of the piston. Meantime, this force generates a torque of 8,940 N·m. This torque will cause a counterclockwise rotation of the reaction mass around the Z-axis. Therefore, the ideal motion of the reaction mass in the Z-direction is disturbed by these motions caused by the oil flow movement.

### 4.1 Analysis of the influence of the horizontal oil inlet on the reaction mass movement

#### 4.1.1 Analysis of simulation results of heavy reaction mass moment

**Fig 7:**
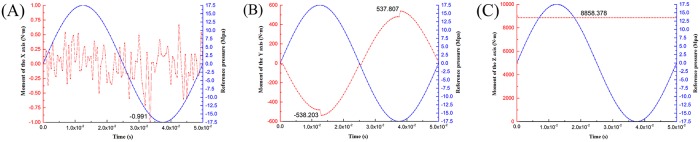
Rotation torque around the X-, Y-, and Z-axis. (A)X (B)Y (C)Z.

This section will examine the rotation torque around the X-axis and Y-axis at different times, and the value of rotation torque around the Z-axis when the pressure reaches the maximum simulation value of the whole cycle in order to plot the change in torque with time and compare it with the reference pressure of the hydraulic chamber. As shown in Fig 7, the blue sinusoid is the reference pressure curve of the hydraulic chamber.

As seen from Fig 7, during the operation of the vibrator, the reaction mass is driven by the X-, Y-, and Z-axis rotation torque. As shown in Fig 7(A), compared with the reference pressure curve of the hydraulic cavity, the rotation torque curve around the X-axis fluctuates irregularly, and the value is very small. The maximum value of the torque is only 0.991 N • m, so the effect on the vibrator can be neglected.

It is shown in Fig 7(B) that the rotational torque curve around the Y-axis is similar to the fluctuation trend of the reference pressure curve of the hydraulic chamber. When the oil enters the upper chamber, the rotation torque around the Y-axis is negative, indicating that the direction of the rotational torque at this moment is clockwise in the direction of the Y-axis; when the oil is injected into the lower chamber, the rotation moment around the Y axis is positive. The direction of the rotational torque at this moment is counterclockwise in the direction of the Y-axis; and when the pressure reaches its maximum value, its value has a certain fluctuation, and its maximum value is 538.203 N·m. In the working process of the vibrator, rotating around the Y-axis torque forces the reaction mass to make periodic pitch movements, and the reaction mass generates a disturbance in the XOZ plane. When the hydraulic pressure reaches the maximum, the moment of rotation is the largest, and the impact on the vibrator is also the largest.

According to Fig 7(C), because the high-pressure channel is always located in the right side of the reaction mass, there is always an eccentric force in the corner. The eccentric force can be equivalent to two forces: one force acting on the reaction mass along the—X-axis and a torque that turns the reaction mass around the Z-axis. Therefore, around the Z-axis, the torque curve changes with time is a constant value, 8858.378 N·m. Meanwhile, the reaction mass’s rotational freedom around the Z-axis is not constrained, resulting the motion deviation of the reaction mass around the Z-axis in the XOY plane and increasing the disturbance of the vibrator. The simulation of rotation torque around the Z-axis is 8858.378 N•m, and the theoretical calculation of torque is 8940.183 N•m. The relative error of the two is 0.915%—it is small. In the range of allowable error, the numerical calculation method is correct and feasible. The simulation force can be calculated by the simulation of torque, and it is 18968.690 N.

From the above analysis, in the vibrator excitation seismic signal process, the reaction mass suffering a force along the—X-axis and torque around the Z-axis is the largest. This force is the main reason for motion disturbance in the XOY plane of the reaction mass. In the next place, the rotation torque around the Y-axis results the pitching motion of the reaction mass, resulting a weak disturbance in the XOZ plane. The moment of rotation around the X-axis has the least influence on the motion of the reaction mass.

#### 4.1.2 Analysis of the influence of the horizontal oil inlet on reaction mass motion

From the above analysis, we can see that the vibrator adopts the method of horizontal oil inlet, which results in the reaction mass being mainly affected by the force along the—X-axis and the rotational torque around the Z-axis. The acting force along the—X-axis is 18968.690 N, resulting in horizontal interference acting on the piston rod by a reaction mass to cause the piston rod to deform. The rotation torque is 8940.183 N▪m around the Z-axis, causing the reaction mass to rotate around the Z-axis, interfering with the vertical vibration of the reaction mass, affecting the trajectory of the reaction mass, and increasing the disturbance of the vibrator. In summary, under the coupled interference of the two forces, the trajectory of the reaction mass is greatly affected, and the disturbance of the vibrator is increased rapidly, which seriously affects the quality of the output signal of the vibrator.

In summary, the ideal vertical motion of the reaction mass is severe disturbed by the force in the negative X-axis as well as the torque produced at the corner of Q as oil flows through the inlet.

### 4.2 Simulation analysis of the piston rod deformation under the horizontal oil inlet

#### 4.2.1 Deformation analysis of piston rod

**Fig 8:**
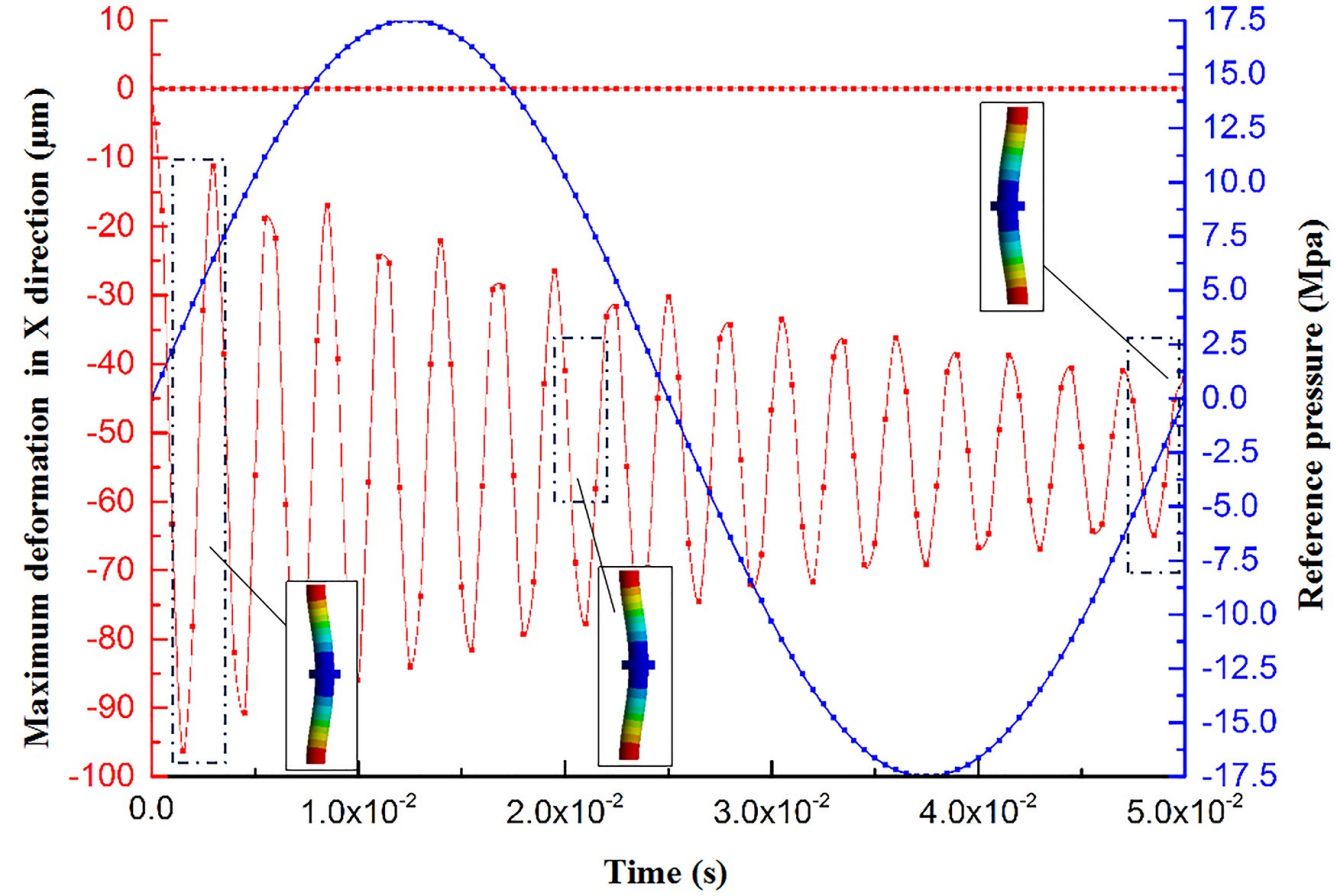
The maximum deformation curve of the piston rod in the X-direction and the deformation nephogram.

This section examines the maximum deformation curve and the deformation nephogram of the piston rod on the direction of X-, Y-, and Z-axis, as shown in Fig 8 and S2 Fig. The red curve is the deformation of the piston rod, the positive value represents positive deformation, and the negative value indicates negative deformation. The blue curve is the reference pressure curve of the hydraulic chamber.

Fig 8 is the curve of maximum deformation with time in the X-direction of the piston rod. Compared with the hydraulic pressure of the hydraulic chamber, the fluctuation trend of the maximum deformation curve of the piston rod along the direction of—X is approximately multicycle weakening, and its fluctuation is much more than that of the reference pressure of hydraulic chamber. The maximum deformation of the + X-direction of the piston rod is smaller than that of the—X-direction, and the curve with time is approximately a horizontal line close to the X-axis. Meanwhile, the piston rod contacts with the reaction mass, and finally, the negative force of the X-axis acts on the piston rod, resulting in a large deformation of the—X-direction of the piston rod. As shown in Fig 8, the piston rod has obvious deformation in the X-direction, and the deformation is mainly concentrated in the middle of the piston rod. Among them, the maximum deformation of the +X-direction of the piston rod is 0.077825 μm, and the maximum deformation of the–X-direction of the piston rod is 96.244 μm. In summary, the piston rod has the deformation of the +X- and -X-direction. The deformation in the direction of—X is the major deformation form, which will produce a disturbance in the X-direction during the operation of the vibrator and force the heavy reaction mass to generate motion disturbance in the XOZ plane.

**Fig 9:**
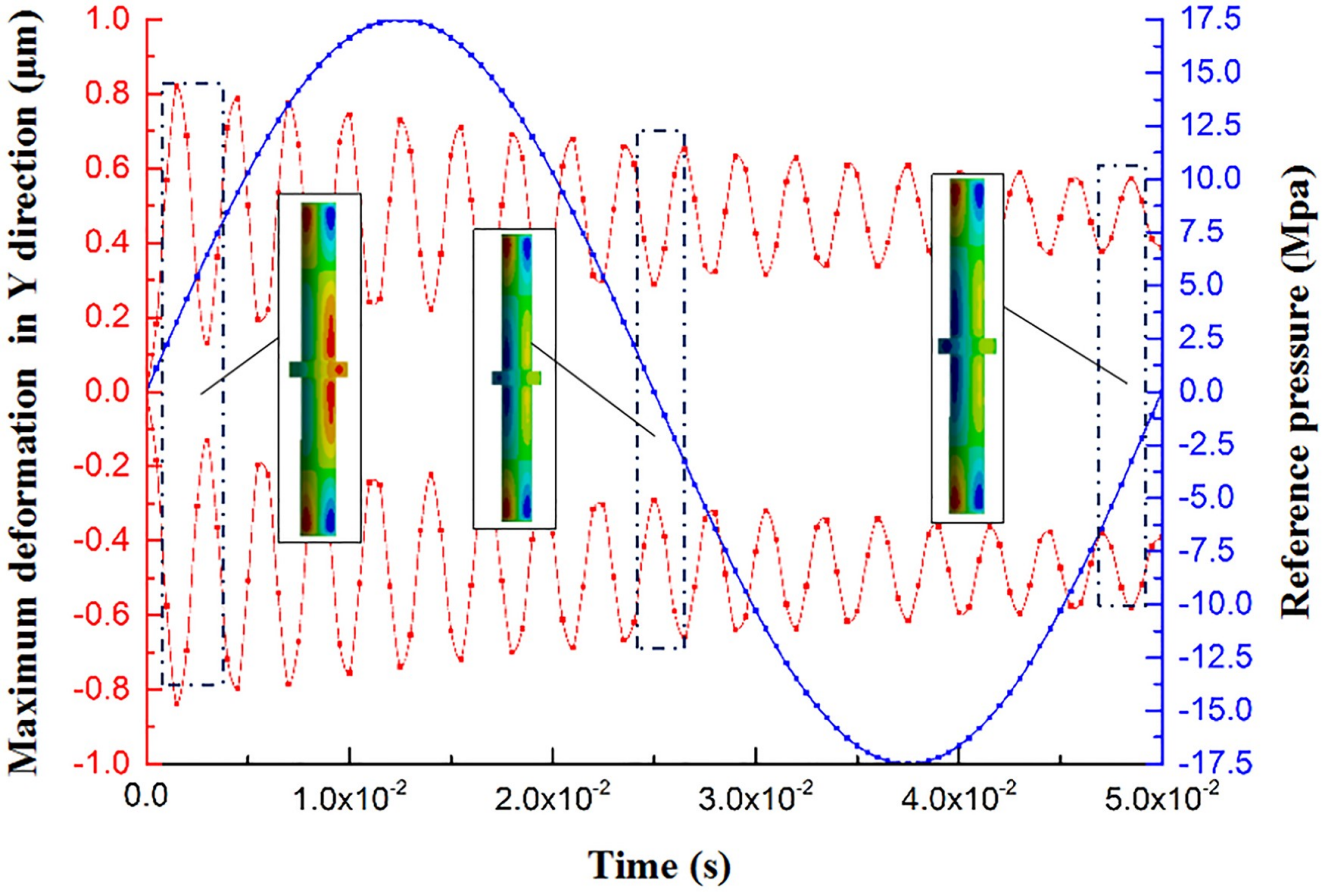
The maximum deformation curve of the piston rod in the Y-direction and the deformation nephogram.

As shown in Fig 9, the deformation analysis is performed compared with the reference pressure, wherein the maximum deformation curves of the piston rod in the +X- and -X-directions are similar, exhibiting a trend of multicycle weakening. However, the target of weakening is not zero. The amount of deformation of the piston rod in the Y-direction is smaller than that of the X-direction. The red area in the figure represents the piston rod having a positive deformation in this part, while the blue area represents a negative deformation in this part. The deformation of the piston rod in the Y-direction mainly occurs in the middle of the piston rod and both ends of the piston rod. Wherein, the +Y-maximum deformation is 0.82002 μm and -Y is 0.83759 μm; the main reason for the deformation of the piston rod in the Y-direction is the difference of the pressure loss and the movement distance when the hydraulic oil reaches the cylinder surface, resulting in uneven hydraulic distribution of the piston rod cylinder surface, will eventually cause the deformation of piston rod. In conclusion, the deformation of the piston rod in the Y-direction will bring in a significant interference in that direction, increasing the motion disturbance of the vibrator reaction mass in the YOZ plane.

**Fig 10:**
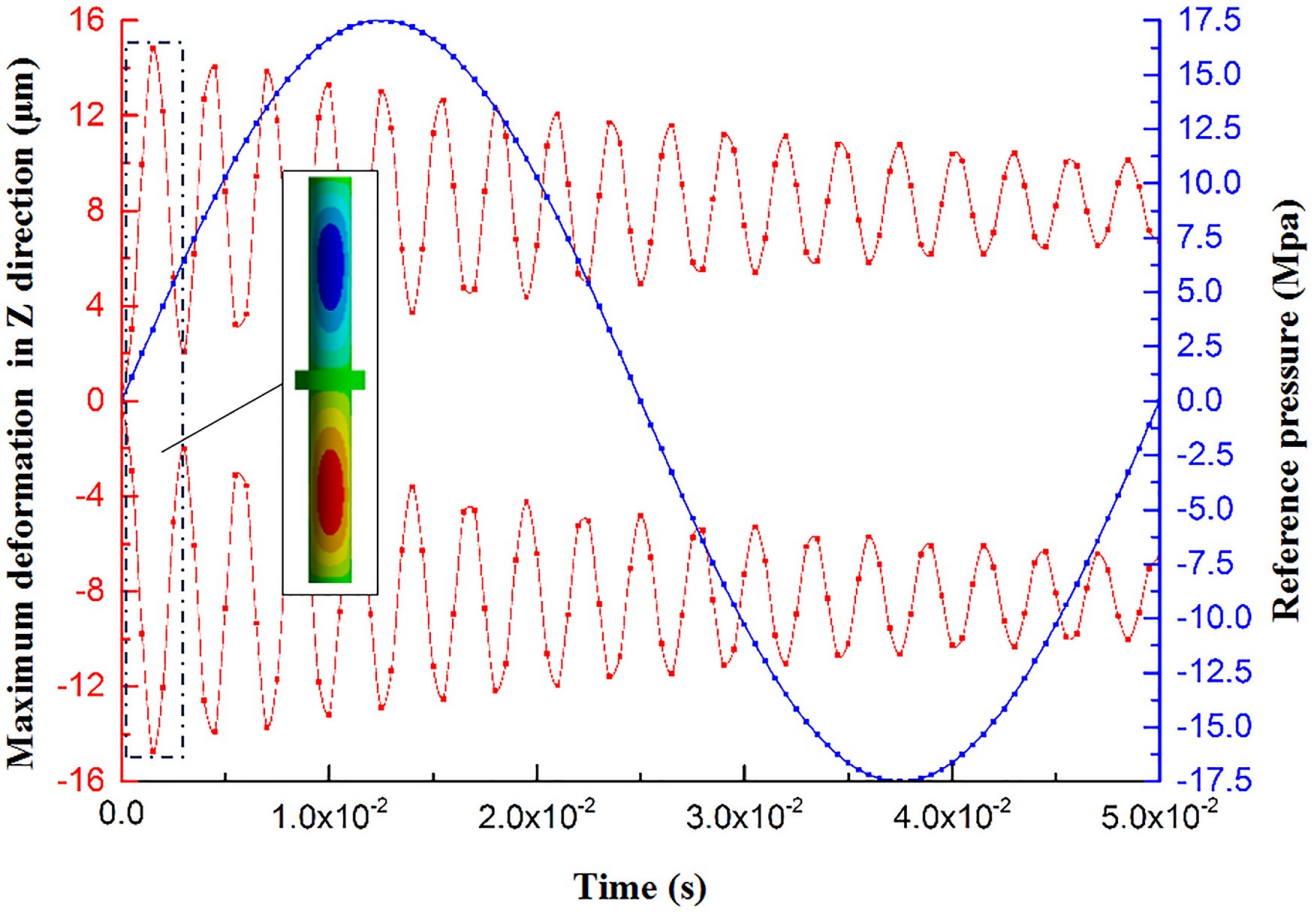
The maximum deformation curve of the piston rod in the Z-direction and the deformation nephogram.

Fig 10 shows the maximum deformation curve of the piston rod in the Z-direction; the analysis result shows that deformation of the piston rod is mainly in the Z-direction at an intermediate position of the two parts of the piston rod. The maximum amount of deformation of the + Z is 14.813 μm, while that of the -Z is 14.732 μm. Since the reaction mass moves along the vertical direction (Z-direction), the Z-direction deformation of the piston rod has very small effects on the reaction mass movement, so the vibrator would not have a disturbing influence.

#### 4.2.2 Analysis of the influence of piston rod deformation on the vibrator disturbance

As seen from the above analysis of the vibrator, the piston rod has a large deformation in the course of vibrator operation; the deformation of the piston rod will drive the reaction mass to move together, causing the vertical interfere of the reaction mass. The trajectory of the reaction mass deviates considerably, significantly increasing the disturbance of the vibrator.

### 4.3 Cause analysis of the vibrator disturbance

In summary, when the vibrator is in operation with a horizontal oil inlet, the hydraulic flow channel is located inside the reaction mass with a three-way asymmetric arrangement, resulting in a coupled interference effect of the reaction mass force along the—X-axis as well as the Z-axis rotation torque; thus, the reaction mass rotates around the Z-axis, forming the reaction mass disturbance. Meanwhile, the piston rod has a certain deformation. The interaction of these two kinds of disturbance drives to form the interference in the X- and Y-direction, affecting the reaction mass trajectory. The trajectory of the test point on the reaction mass is a space curve, increasing the disturbance of the vibrator and seriously affecting the quality of the output signal of the vibroseis. Therefore, the use of a horizontal oil inlet is a direct cause of vibrator disturbance.

## 5 Research of the disturbance control for the vibrator

### 5.1 Vertically oriented oil inlet of the vibrator

**Fig 11:**
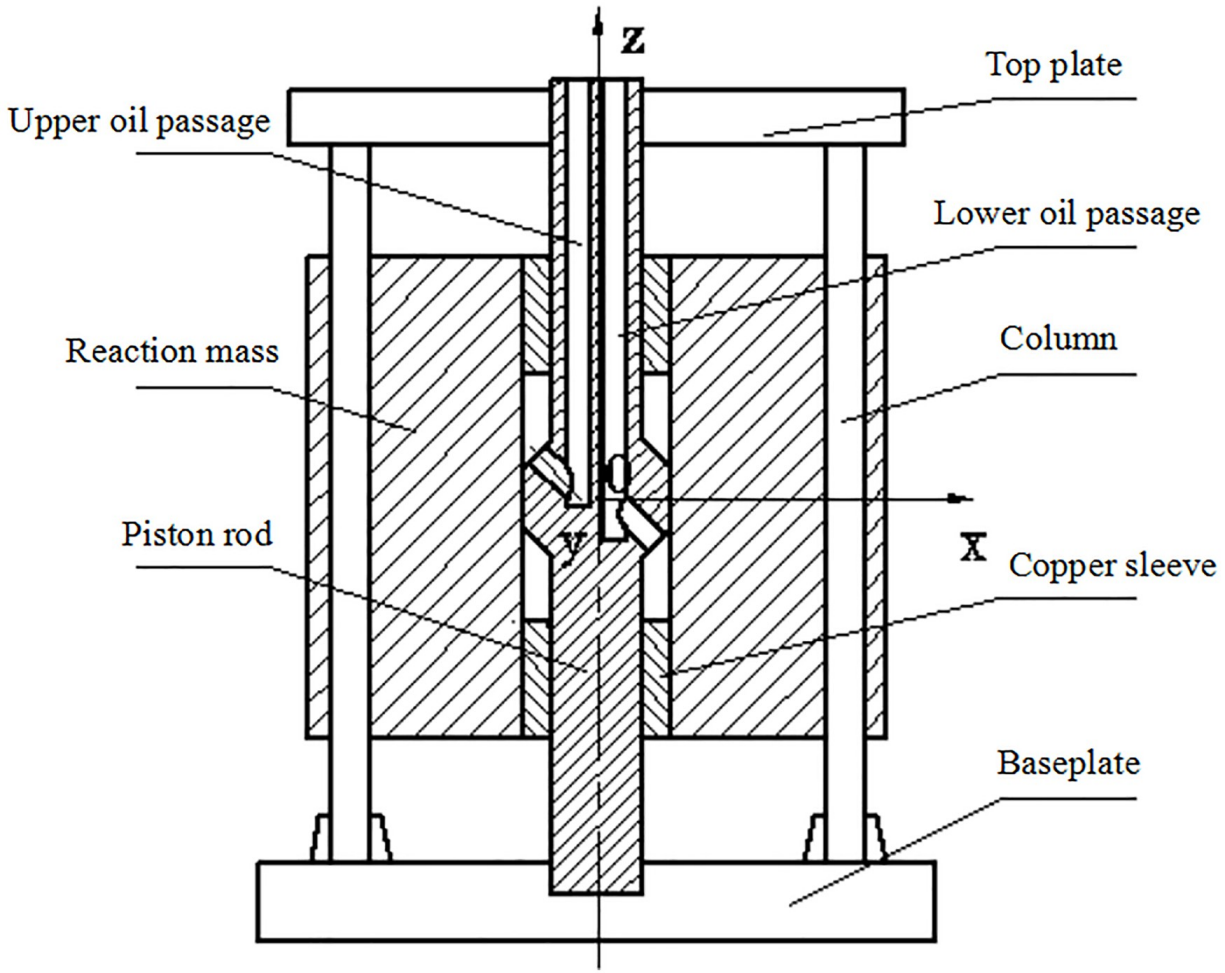
Simple structure of the vertical passageway vibrator.

The horizontal oil inlet causes the disturbance mentioned above. To solve this problem caused by inlet orientation, there is another way to input oil to the vibrator: the hydraulic runner is vertically oriented inside the piston rod, as shown in Fig 11.

### 5.2 Simulation and boundary conditions

**Fig 12:**
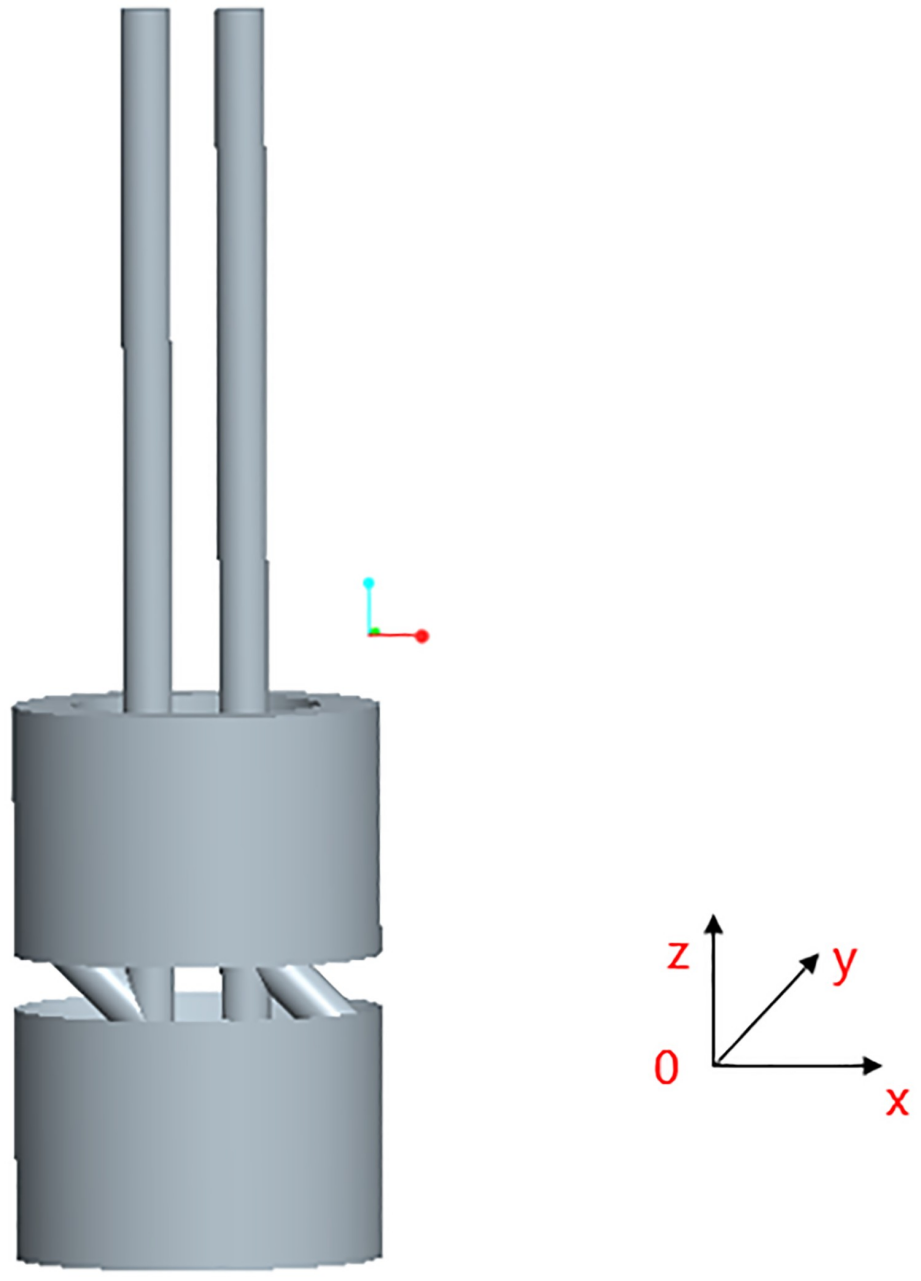
Fluid domain model of the vertical oil passageway.

**Fig 13:**
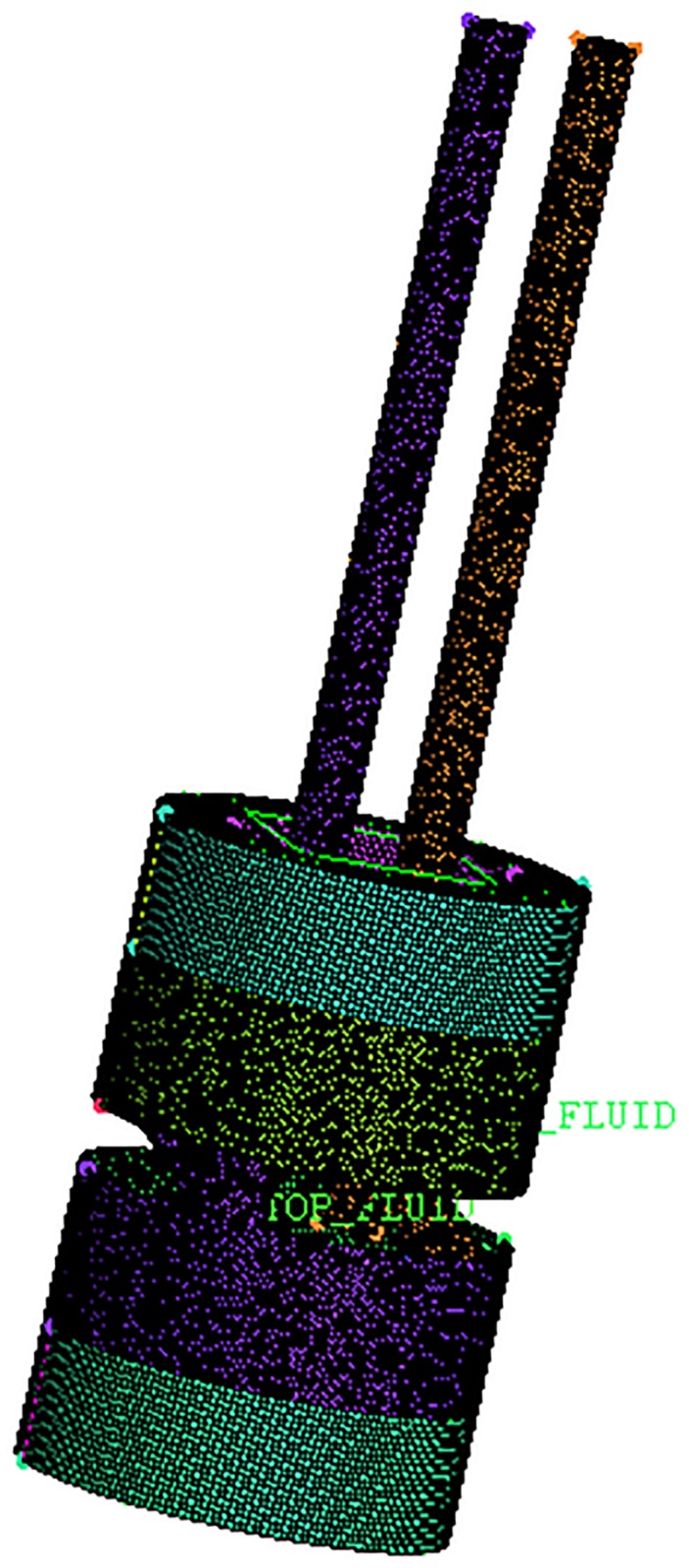
Meshing.

**Fig 14:**
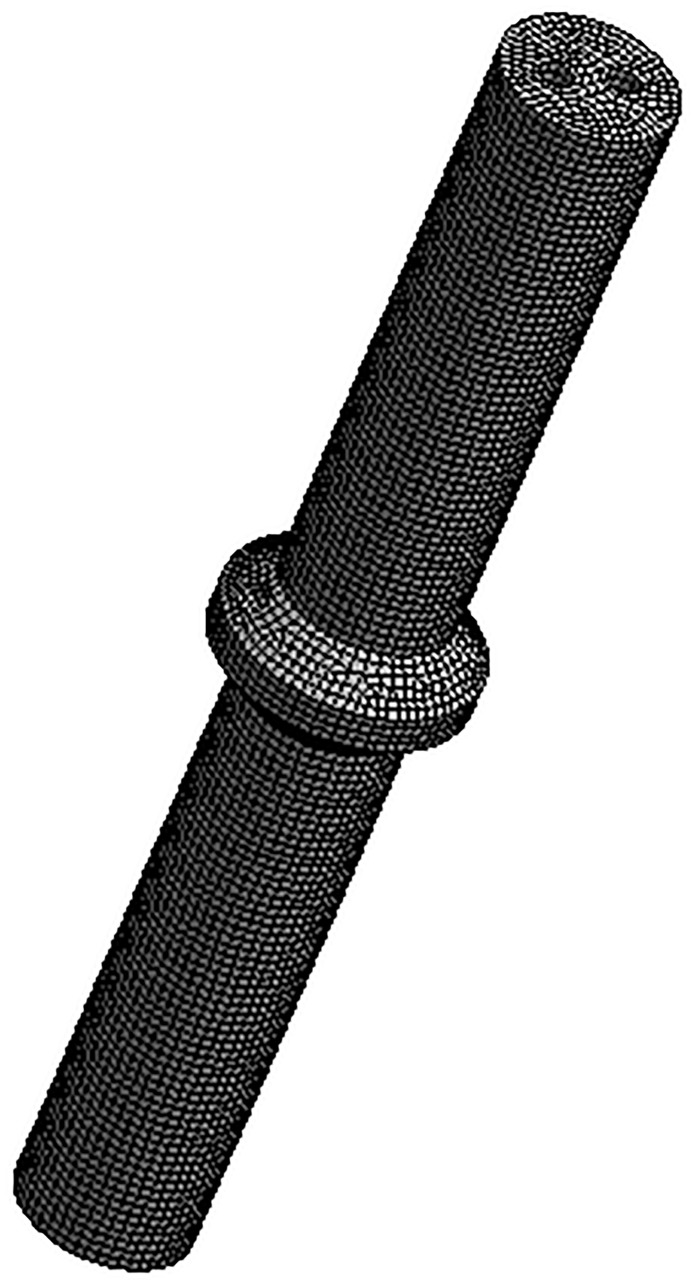
Geometric model of piston rod.

**Fig 15:**
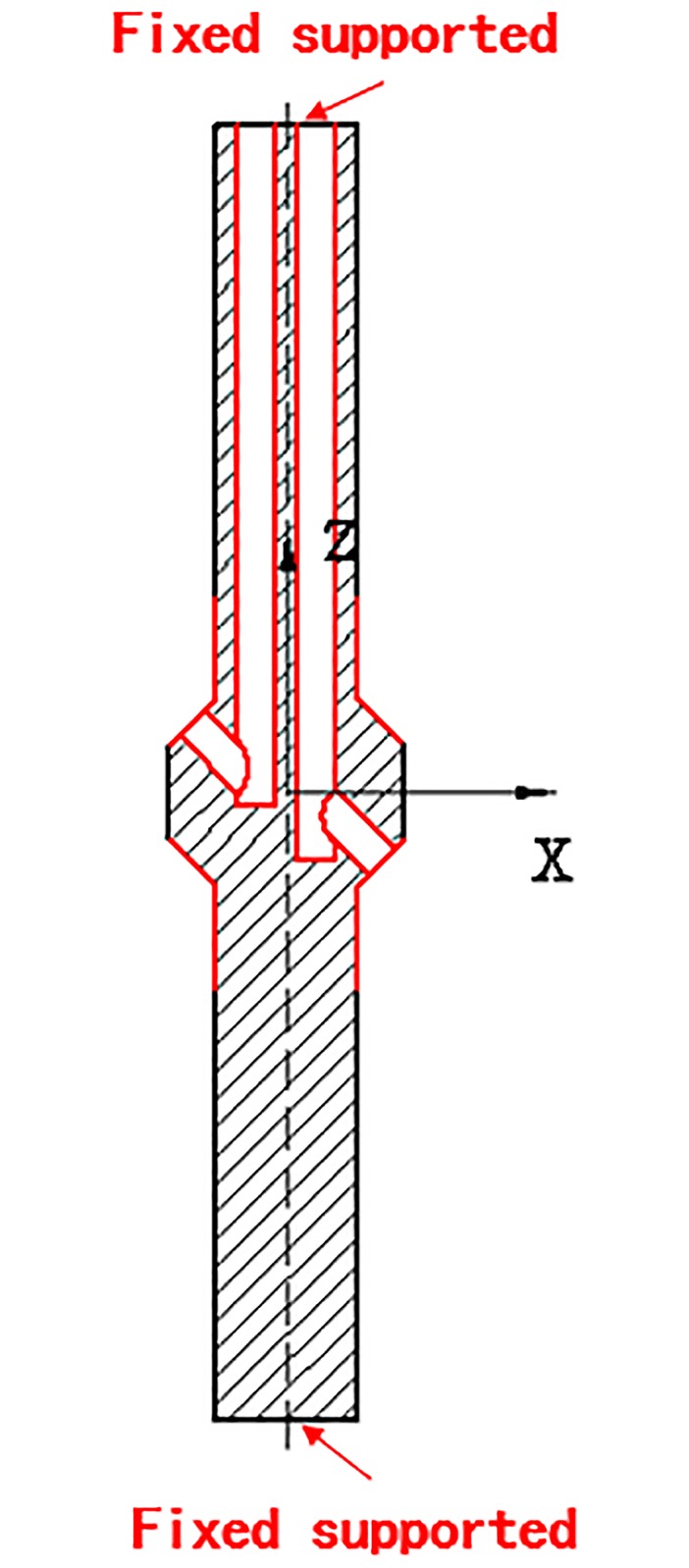
Boundary condition of the piston rod.

The hydraulic flow path geometry model and simulation model of the piston rod are established, as shown in Figs 12, 13, 14 and 15, and the spatial Cartesian coordinate system O-XYZ is set up.

### 5.3 Results of the simulation between two oil inlet methods

**Fig 16:**
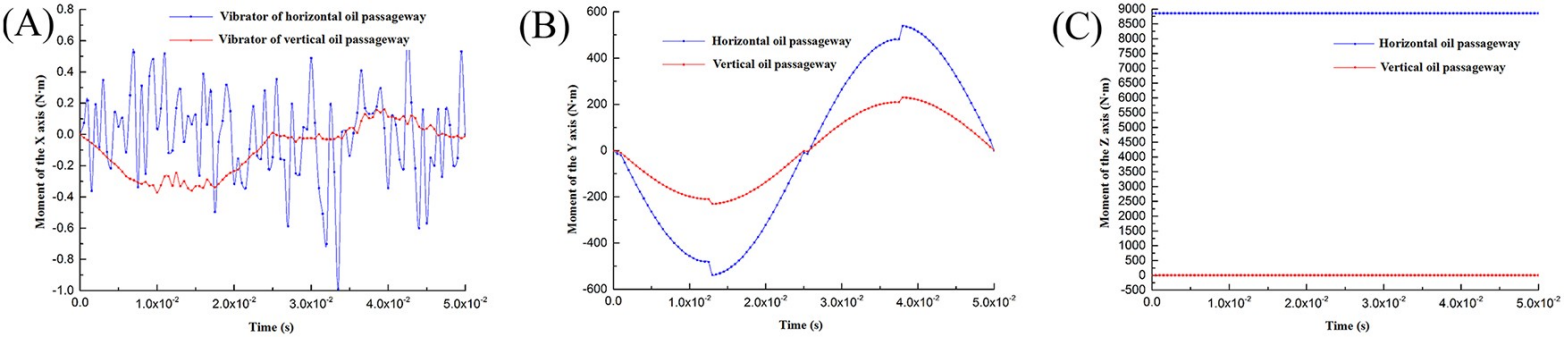
Comparison of two passageway rotation torque. (A) X-axis (B) Y-axis (C) Z-axis.

**Fig 17:**
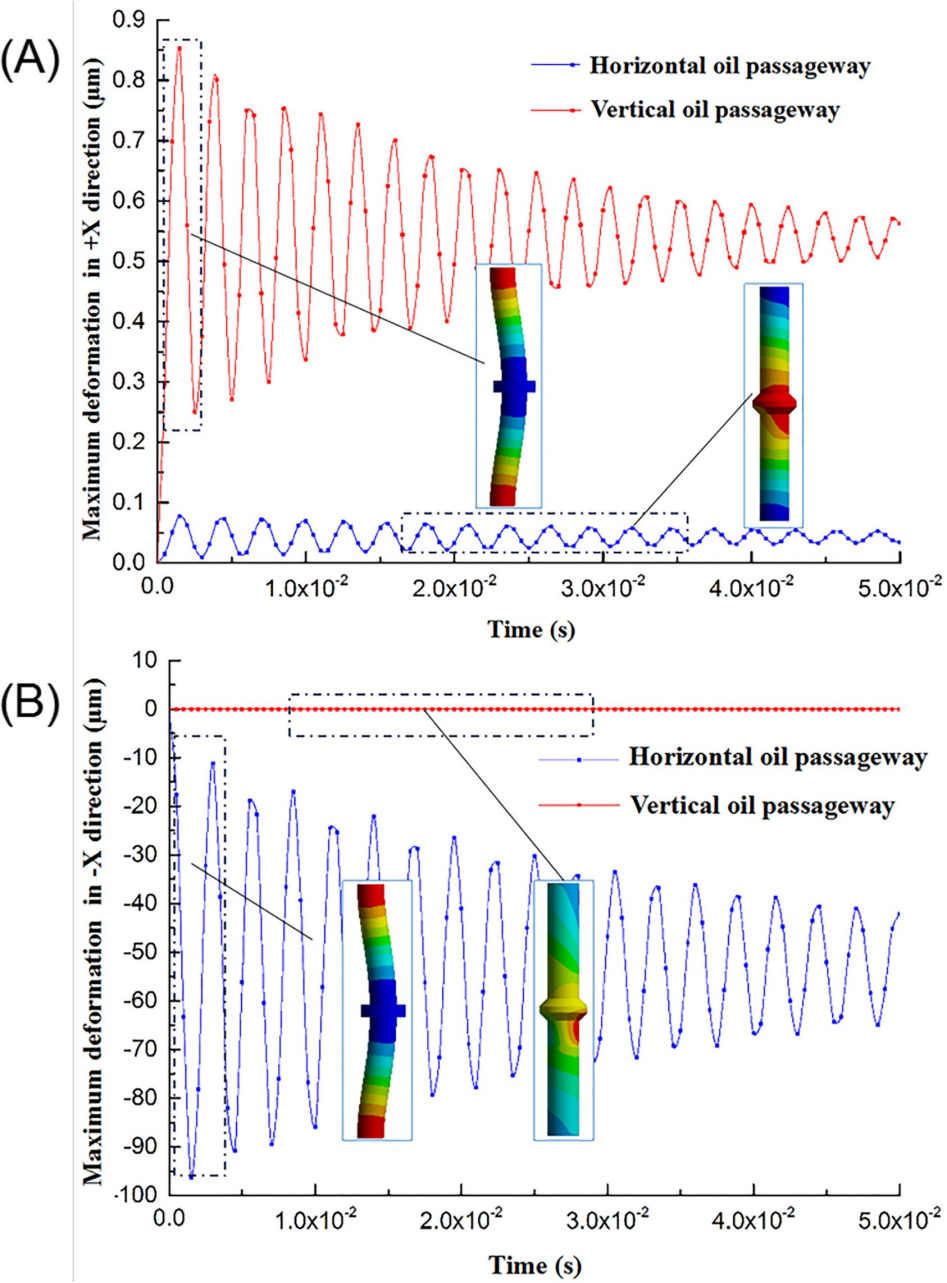
Comparison of piston rod X-direction deformation. (A) Direction of +X (B) Direction of -X.

**Fig 18:**
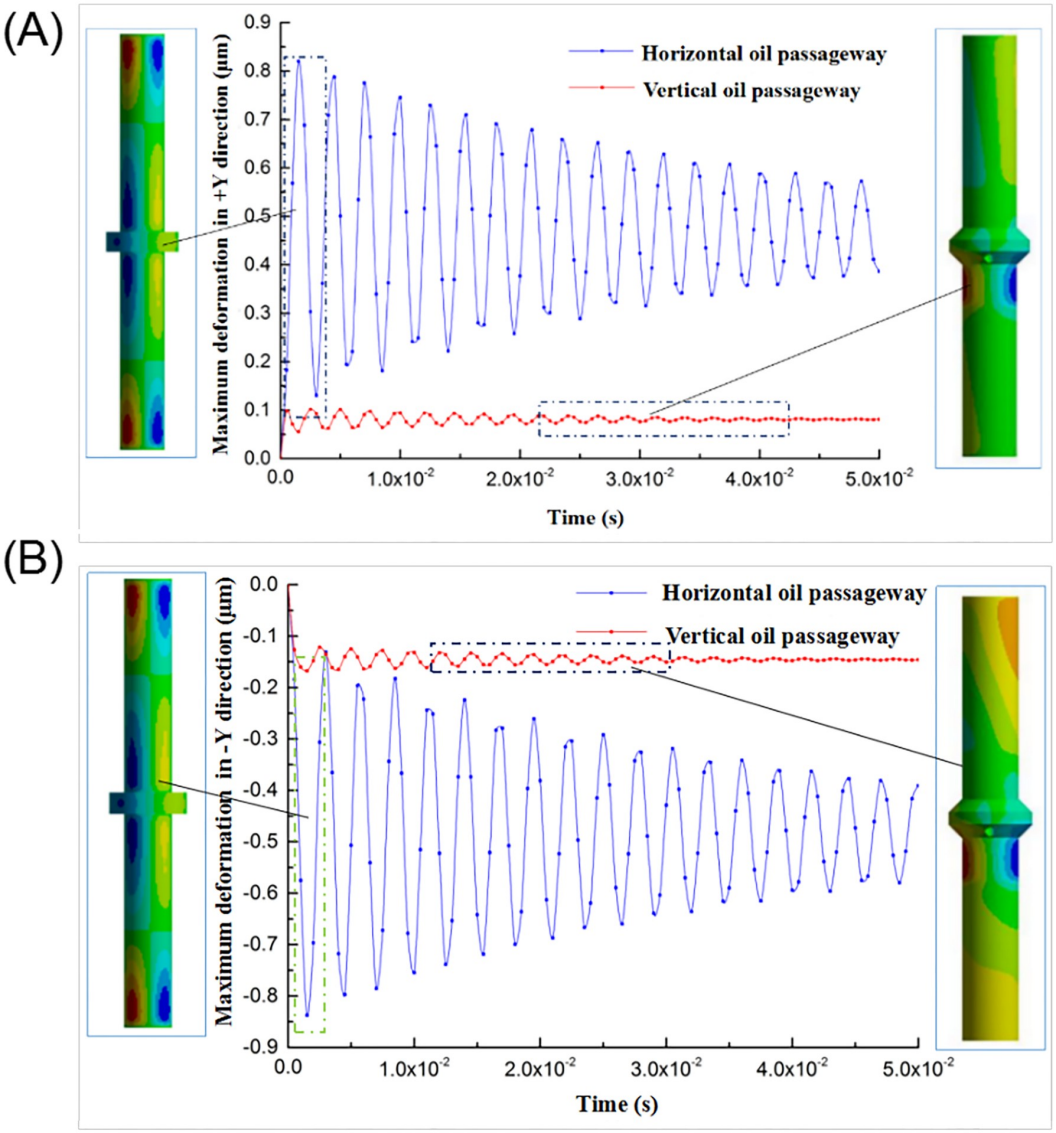
Comparison of piston rod Y-direction deformation. (A) Direction of +Y (B) Direction of -Y.

1. Comparison of the reaction mass torque.
  The vertical inlet method is used to simulate the same setting as the vibrator in the horizontal inlet mode, and the simulation results are extracted and compared with the results of the horizontal one, as shown in Figs 16, 17 and 18.
  As shown in Fig 16(A), the rotation torque around the X-axis of vertical oil inlet is smaller than that of the horizontal inlet, and the fluctuation range is small at the same time; therefore, the effects on the reaction mass movement can be neglected because the two results of the fluctuation range are very small.
  As seen from Fig 16(B), compared to horizontal method, the rotational torque around the Y-axis of the vertical inlet method is much lower than that of the horizontal method; since the hydraulic flow path is arranged inside the piston rod, the rotational moment will not have an impact on the reaction mass movement because it acts on the piston rather than on the reaction mass.
  In Fig 16(C), the rotational torque around the Z-axis generated by the horizontal inlet mode has a great influence on the reaction mass, up to 8858.378 N·m. The simulation result of the vertical inlet mode is approximately a straight line with Y = 0. Therefore, the hydraulic flow channel will not make the reaction mass rotate around the Z-axis when the oil inlet is vertical.
2. Comparison of piston rod deformation.
  It can be seen from Fig 17(A) that the deformation and fluctuation amplitude of the piston rod in the horizontal direction are smaller than that of the vertical. However, it can be seen from Fig 17(B) that the amount of negative deformation of the piston rod in the X-direction is much smaller for the vertical oil inlet than it is for the horizontal one. In addition, the displacement of the piston rod in the X-direction to the equivalent total deformation is less than that of the amount of the horizontal inlet piston rod deformation; under the use of the vertical oil inlet method, the piston rod’s X-direction deformation is significantly reduced.
  It can be seen from Fig 18(A) and 18(B) that if the oil inlet is oriented vertically, the amount of deformation of the piston rod in the +Y- and—Y-direction would be smaller than that of the horizontal method, and the fluctuation amplitude would also be slower. Therefore, by using the vertical oil inlet vibrator, the impact of piston rod deformation in the Y-direction on the vibration disturbance could be greatly reduced.
  In summary, due to the use of the vertical oil inlet method, the hydraulic flow channel will not produce a very large torque and deformation of the piston rod; it also significantly reduces the reaction mass movement interference caused by the method of oil feeding, and the vibrator disturbance is significantly reduced.

## 6. Experimental tests and results

**Fig 19:**
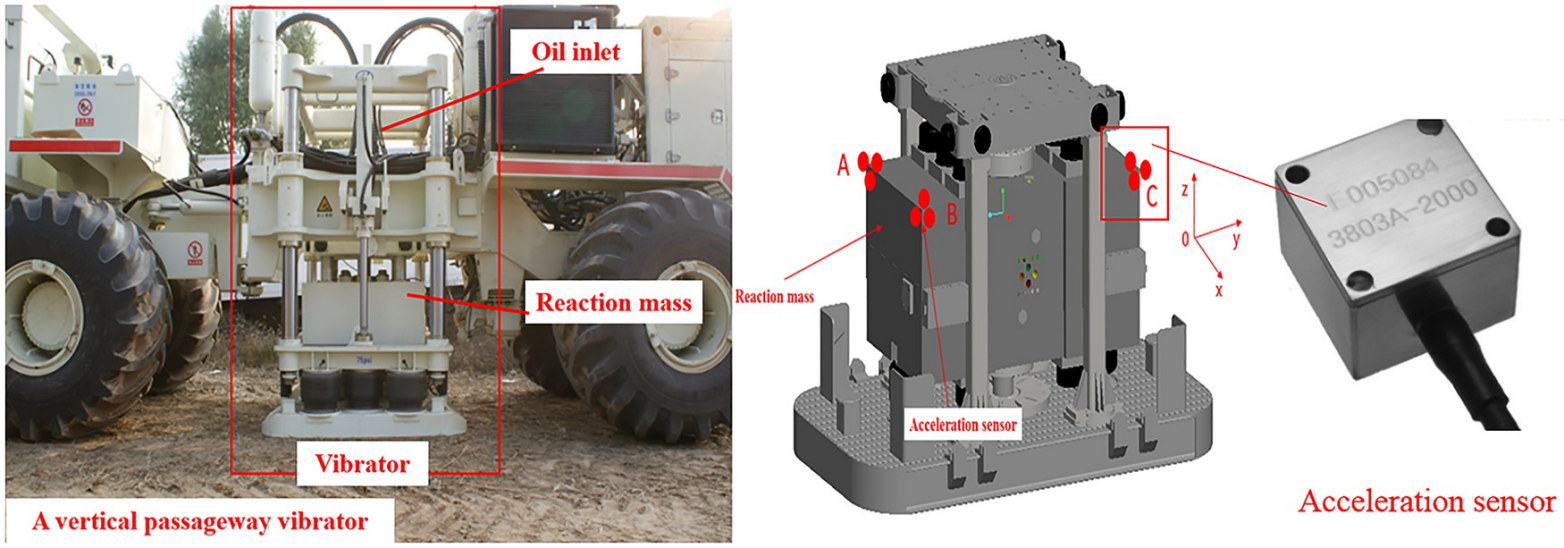
Layout of the testing points for the reaction mass.

To demonstrate the findings from model simulations, an experiment is carried out. Fig 19 shows a testing setup. In total, nine accelerometers are placed at three vertices of the reaction mass. These vertices are marked as A, B and C. At each vertex, three accelerometers are positioned to detect the motions in the X, Y and Z directions, and the test process can be divided into three steps.

### 6.1 Equipments and materials

The vertical oil inlet mode disturbance test of the vibrator is carried out in the vibroseis test field. The equipment used in the tests includes vibrator, acceleration sensor (accelerometers), excitation control box, NI instruments, software LabVIEW and cables. The size of the vibrator reaction mass is 1080×1080×680 mm.

### 6.2 Test process

**Fig 20:**
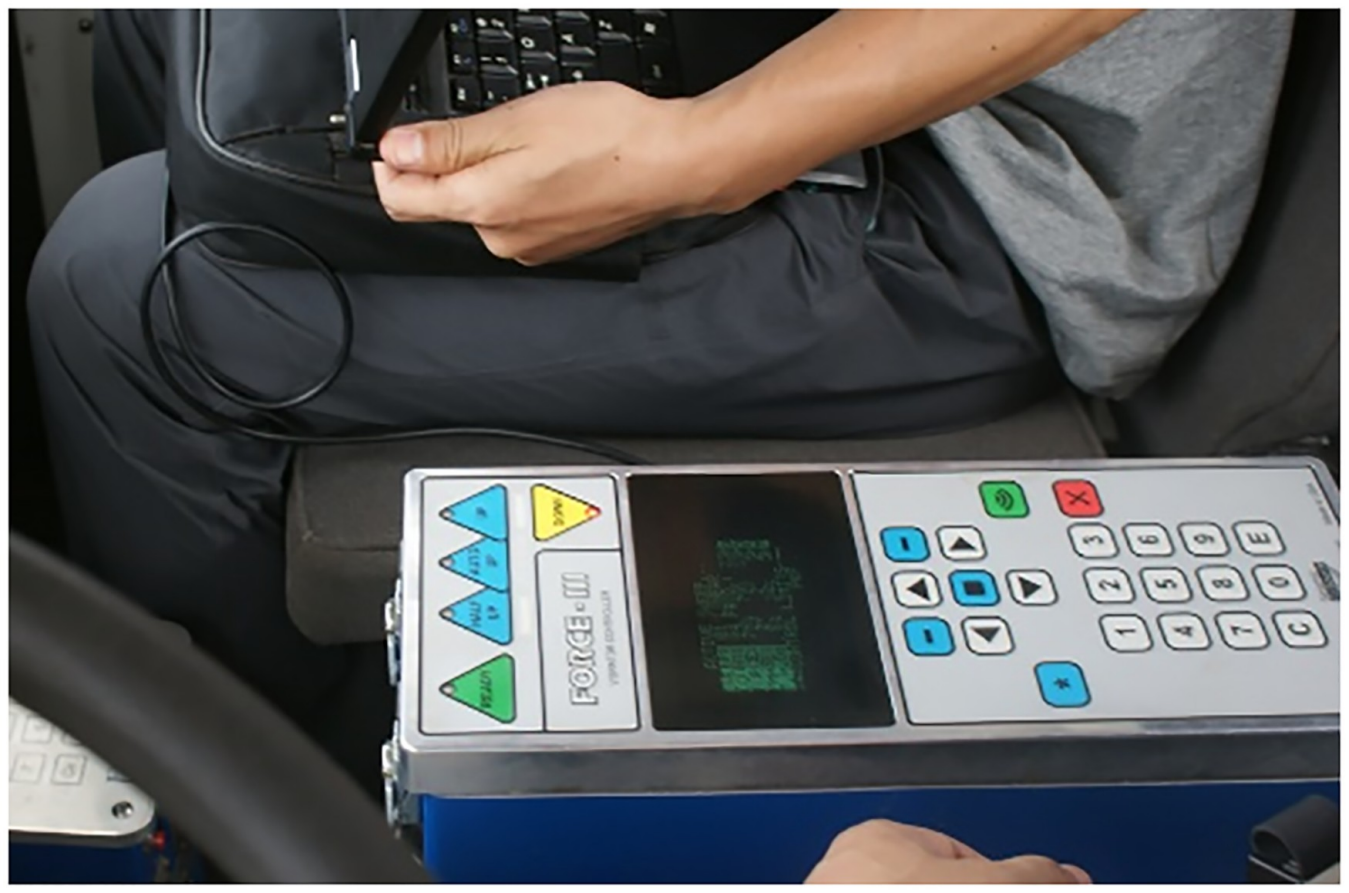
Excitation control box.

**Fig 21:**
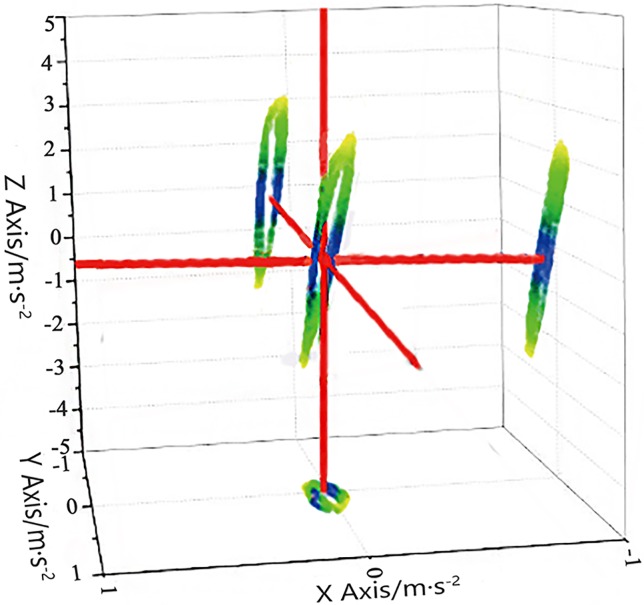
A reaction mass point actual movement path of vertical passageway.

1. Layout of test equipment. As shown in Fig 19, the accelerometers were arranged at three vertices of the vibrator reaction mass for detecting the acceleration in two orthogonal directions in the vertical and the horizontal planes.
2. The acquisition of test results. During the operation of the vibrator, the seismic wave excitation of the vibrator is controlled by the excitation control box (Fig 20). The acceleration sensor detected the acceleration of the points and transmitted it to LabVIEW through NI instruments, and the accelerations in the two orthogonal directions (vertical and horizontal plane) were combined by the LabVIEW at each sampling time. Then, all the acceleration samples in the form of a synthesis vector were connected to obtain the acceleration curve of the testing point. According to the acceleration curve of the three vertices, the reaction mass disturbance curve was obtained.
3. Analysis of test results. The disturbance law of the vibrator could be obtained from the reaction mass disturbance curve. As shown in Fig 21, in the vertical oil inlet method, the trajectory deviation away from the designed trace has been reduced significantly compared with that of the horizontal method. The test results showed that the disturbance of the vibrator in the X- and Y-directions was reduced, which matches well with the simulation results. Therefore, the vibrator with a vertical oil inlet method could effectively reduce the interference with the vibrator in the X- and Y- direction, significantly reducing the vibrator disturbance and contributing to a high-quality signal excitation.

### 6.3 Test results

**Fig 22:**
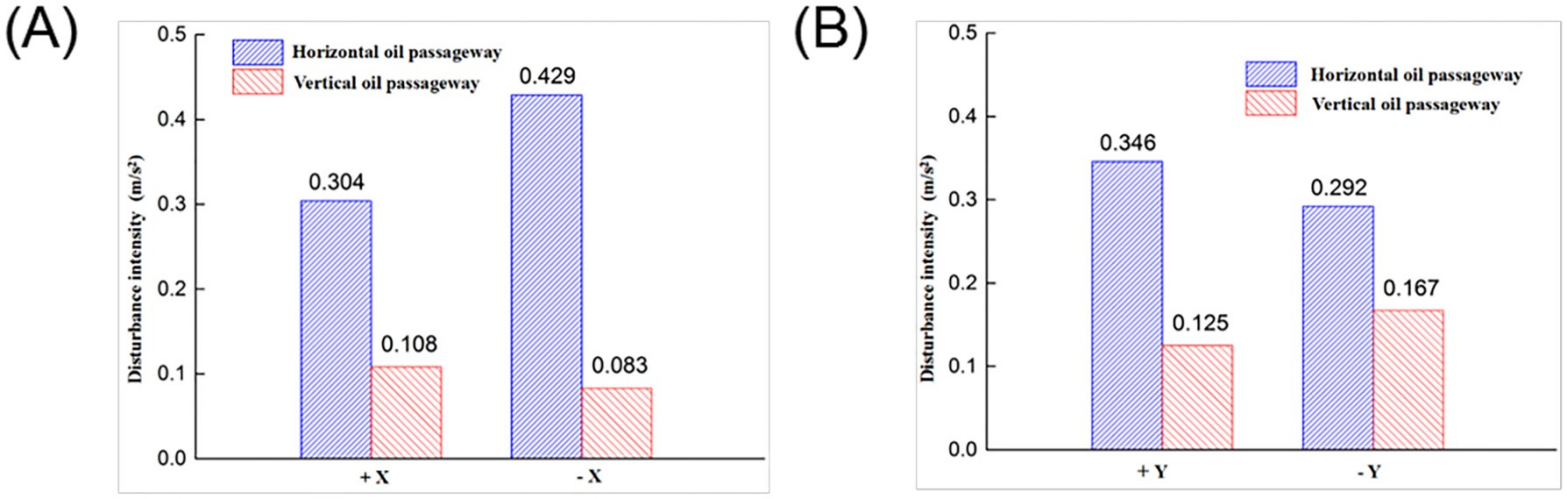
Comparison of the disturbance intensity between the horizontal and vertical oil passageways. (A) Disturbance intensity of the X-axis (B) Disturbance intensity of the Y-axis.

According to Figs 19, 20 and 21, a further comparative analysis of vibrator disturbance was carried out by examining the disturbance value of the two kinds of oil inlet geometries. As shown in Fig 22, the blue shaded bar is the horizontal oil inlet mode, and the red shaded bar is the vertical oil inlet mode.

As shown in Fig 22, it can be seen that the disturbance value of +X and -X was reduced by 64.474% and 80.653%, respectively, and the value of disturbance in the directions of +Y and -Y was reduced by 63.873% and 42.808%, respectively. In addition, the absolute values of disturbance in the direction of X and Y, the disturbance amplitude, could be obtained, as shown in Table 1.

**Table 1:** Comparative analysis of disturbance amplitude with two oil inlet methods.

Oil inlet method	Disturbance amplitude in the X-direction /m·s-2	Disturbance amplitude in Y-direction /m·s-2
Horizontal	0.733	0.638
Vertical	0.191	0.292
Suppressed ratio	73.943%	54.232%

As shown in Table 1, the disturbance amplitudes of the vertical method in the directions of X and Y were suppressed by 73.943% and 54.232% compared with those of the horizontal method.

In summary, there is a large X- and Y-direction disturbance using the horizontal oil inlet method. The disturbance amplitude for the X- and Y-directions could be reduced by 73.943% and 54.232%, respectively, after using the vertical method, indicating that the interference of the horizontal vibration has been effectively suppressed, so the vibrator disturbance is reduced. The main reason is that the use of the vertical oil inlet method could significantly reduce the piston rod radial force and the reaction mass rotation torque. The piston rod deformation and reaction mass rotational movement disappeared, which effectively suppressed the horizontal disturbance, leading to a very large improvement in the vibrator performance.

## 7 Conclusions

1. The high-frequency excitation signal of the vibrator is characterized by an obvious signal distortion and low signal-to-noise ratio. The hydraulic disturbance of the vibrator under the horizontal oil inlet mode is the main reason for the high distortion and disturbance of the geophysical signal.
2. The reason for the hydraulic disturbance in the method of the horizontal oil inlet mode has been analyzed. ① The hydraulic channel arranged inside the reaction mass is asymmetrical in three directions, resulting in the coupling disturbance of the negative force acting along the X-axis and the rotation torque around the Z-axis. This disturbance will lead to a hydraulic disturbance of the reaction mass in the XOY plane. ②Meanwhile, the piston rod has a deformation along the X- and Y-directions, causing the motion deviation of the reaction mass and a hydraulic disturbance in the XOZ and YOZ planes. All these factors made the vibrator disturbance increase rapidly, finally leading to the low signal-to-noise ratio and high distortion of the output geophysical signal.
3. After analysis of the technique suppressing the reaction mass disturbance, the vertical oil inlet method is adopted, the heavy reaction mass is no longer affected by the rotational torque, and the deformation of the piston rod is greatly reduced, which reduces the interference of the vibrator in the X- and Y-directions. Combined with the vertical oil inlet mode disturbance test, it is shown that the vibrator disturbance in the X- and Y-directions is decreased by 73.943% and 54.232%, respectively. The vibrator hydraulic disturbance is weakened, bringing a significant improvement for stimulating a high-quality, high-resolution geophysical signal.
4. Therefore, the vertical oil inlet method of the reaction mass is strongly recommended in the vibrator system. Furthermore, the oil inlets angle of the piston rod should be furtherly researched and optimized, in order to reduce the hydraulic disturbance, and improve the geophysical signal.

## Supporting information

S1 File The data of the vibrator hydraulic oil pressure. (PDF)

S2 File The data of the piston rod deformation. (PDF)

S1 Fig The setting of vibrator hydraulic loading (A) the loading curve of the hydraulic oil (B) UDF for hydraulic force. (PDF)

S2 Fig The coordinates of the piston rod deformation analysis. (PDF)
