# Supplementary material for: Disturbance study of seismic vibrator reaction mass and piston
Source: PLoS One. 2019 Dec 5;14(12):e0225259. doi: 10.1371/journal.pone.0225259 (PMC6894776; doi:10.1371/journal.pone.0225259)
Supplement: S1 File — (PDF) [file pone.0225259.s001.pdf]

The data of the Vibrator hydraulic oil pressure

The hydraulic oil is alternately fed into the upper and lower chambers formed by the reaction mass and the piston rod, under the control of the servo valve. The hydraulic oil pressure is expressed as 17.5sin2πft MPa. It is also shown in Fig. 1. Wherein, *f* is the working frequency, 20 Hz. In the positive half cycle (0-0.025 s), the positive pressure represents the upper chamber oil inlet. In the negative half cycle (0.025-0.05 s), the negative pressure represents the lower chamber oil inlet.

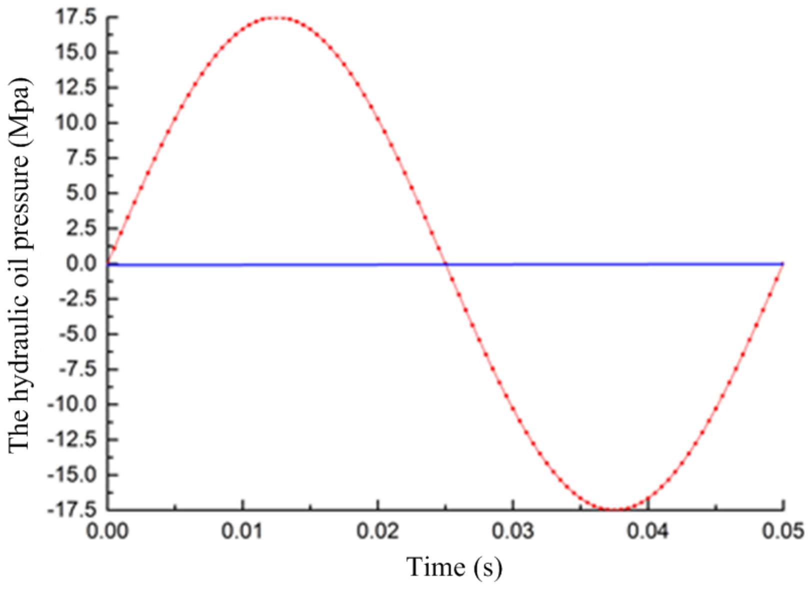

Fig.1 The hydraulic oil pressure change curve

In order to realize the reversing function of the servo valve in simulation analysis, the boundary conditions are redeveloped. UDF files for the inlet and outlet of the hydraulic passageway are compiled, as shown in Fig 2.

```
#include "udf.h"
DEFINE_PROFILE(pressure_inlet,t,nv)
{
   face_t f;
   real time=CURRENT_TIME;
   begin_f_loop(f,t)
  {
   if(time<=0.025)
   F_PROFILE(f,t,nv)=17.5e6*sin(6.2832*20*time);
   else
   F_PROFILE(f,t,nv)=6.2e5;
  }
   end_f_loop(f,t)
}
```

```
#include "udf.h"
DEFINE_PROFILE(pressure_outlet,t,nv)
{
   face_t f;
   real time=CURRENT_TIME;
   begin_f_loop(f,t)
  {
   if(time<=0.025)
   F_PROFILE(f,t,nv)=6.2e5;
   else
   F_PROFILE(f,t,nv)=-17.5e6*sin(6.2832*20*time);
  }
   end_f_loop(f,t)
}
```

Fig.2 The UDF programs of the oil inlet boundary conditions
