# Supplementary material for: Disturbance study of seismic vibrator reaction mass and piston
Source: PLoS One. 2019 Dec 5;14(12):e0225259. doi: 10.1371/journal.pone.0225259 (PMC6894776; doi:10.1371/journal.pone.0225259)
Supplement: S2 File — (PDF) [file pone.0225259.s002.pdf]

The data of the piston rod deformation

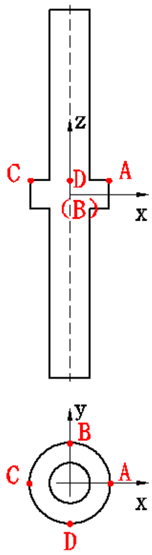

Fig.3 Key points distribution in piston rod

Table.1 The deformation value of the key points of the piston rod

Location	Peak wave value /μm			Trough value /μm	
A	54.019	54.015	54.031	53.807	53.781
B	54.379	54.376	54.391	54.166	54.141
C	53.912	53.909	53.925	53.701	53.675
D	54.379	54.375	54.391	54.166	54.140
Average	54.172	54.169	54.181	53.960	53.934
