# Supplementary material for: Disturbance study of seismic vibrator reaction mass and piston
Source: PLoS One. 2019 Dec 5;14(12):e0225259. doi: 10.1371/journal.pone.0225259 (PMC6894776; doi:10.1371/journal.pone.0225259)
Supplement: S1 Fig — (PDF) [file pone.0225259.s003.pdf]

A

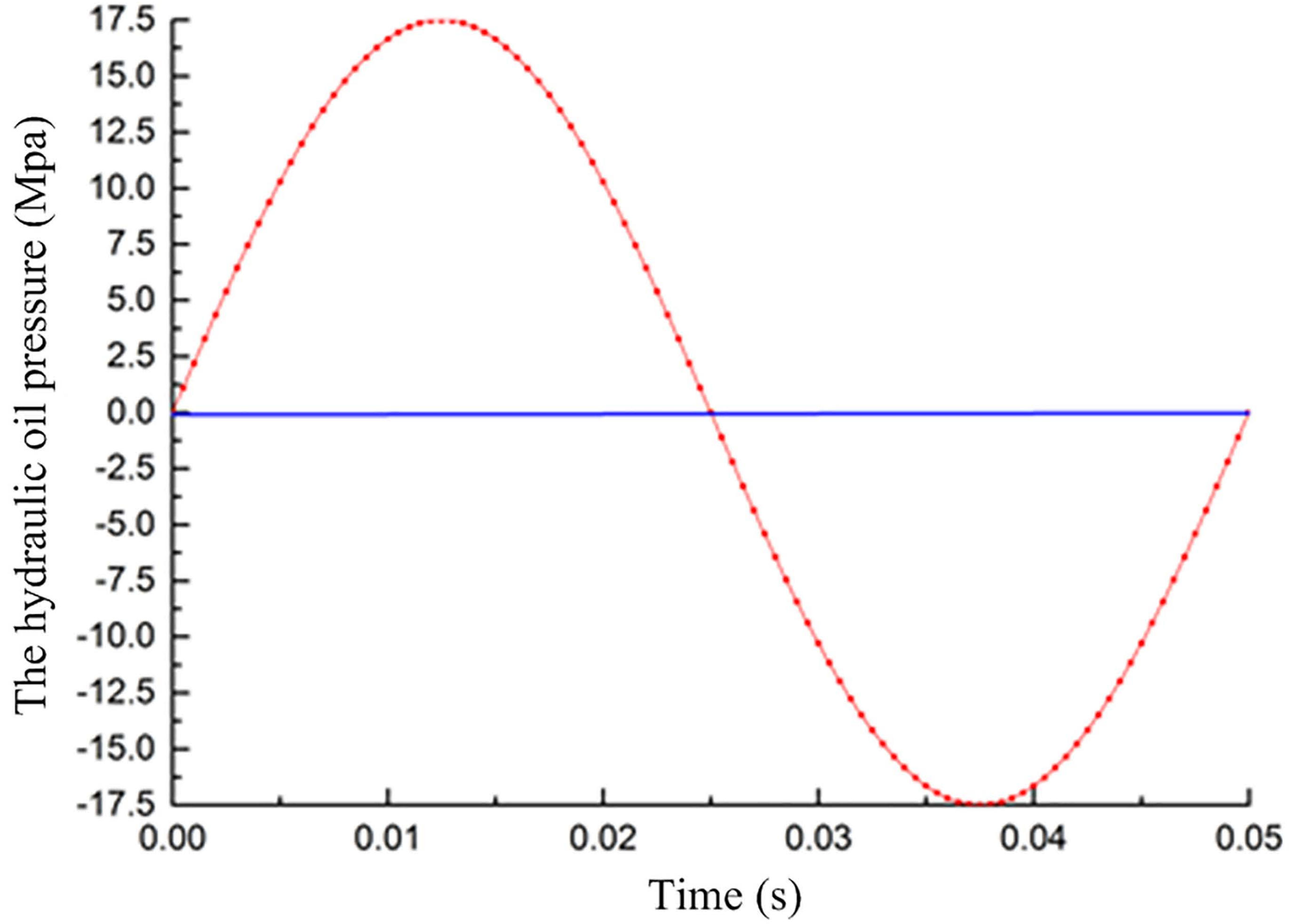

B

```
#include "udf.h"
DEFINE_PROFILE(pressure_inlet,t,nv)
{
   face_t f;
   real time=CURRENT_TIME;
   begin_f_loop(f,t)
  {
   if(time<=0.025)
   F_PROFILE(f,t,nv)=17.5e6*sin(6.2832*20*time
   else
   F_PROFILE(f,t,nv)=6.2e5;
  }
   end_f_loop(f,t)
}
```

```
#include "udf.h"
DEFINE_PROFILE(pressure_outlet,t,nv)
{
   face_t f;
   real time=CURRENT_TIME;
   begin_f_loop(f,t)
  {
   if(time<=0.025)
   F_PROFILE(f,t,nv)=6.2e5;
   else
   F_PROFILE(f,t,nv)=-17.5e6*sin(6.2832*20*time);
  }
   end_f_loop(f,t)
}
```
