# Supplementary figures and images for: Disturbance study of seismic vibrator reaction mass and piston
Source: PLoS One. 2019 Dec 5;14(12):e0225259. doi: 10.1371/journal.pone.0225259 (PMC6894776; doi:10.1371/journal.pone.0225259)

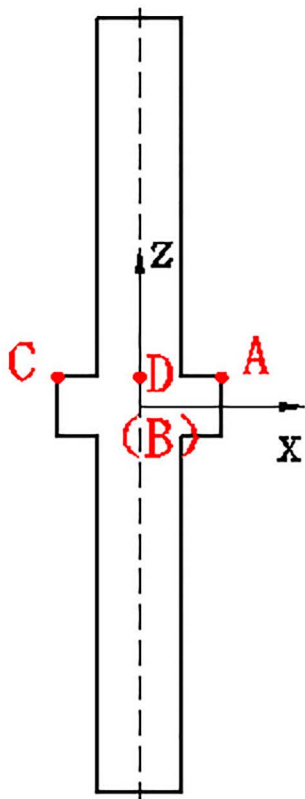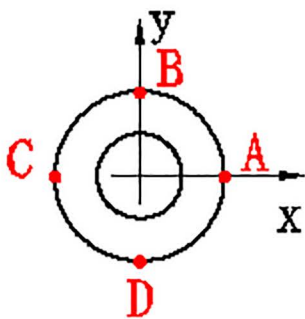

Supplement: S2 Fig — (PDF) [file pone.0225259.s004.pdf]
